# Normal Table of *Xenopus* development: a new graphical resource

**DOI:** 10.1242/dev.200356

**Published:** 2022-07-14

**Authors:** Natalya Zahn, Christina James-Zorn, Virgilio G. Ponferrada, Dany S. Adams, Julia Grzymkowski, Daniel R. Buchholz, Nanette M. Nascone-Yoder, Marko Horb, Sally A. Moody, Peter D. Vize, Aaron M. Zorn

**Affiliations:** 1 www.natalya.com, Cambridge, MA, USA; 2 Xenbase, Division of Developmental Biology, Cincinnati Children's Hospital Research Foundation, 3333 Burnet Ave, Cincinnati, OH 45229, USA; 3 Lucell Diagnostics Inc, 16 Stearns Street, Cambridge, MA 02138, USA; 4 Department of Molecular Biomedical Sciences, College of Veterinary Medicine, North Carolina State University, Raleigh, NC 27695, USA; 5 Department of Biology Sciences, University of Cincinnati, Cincinnati, OH 45221, USA; 6 National Xenopus Resource, Marine Biological Laboratory, Woods Hole, MA 02543, USA; 7 Department of Anatomy and Cell Biology, George Washington University Medical Center, Washington, DC 20037, USA; 8 Xenbase, Department of Biological Science, University of Calgary, Calgary, Alberta T2N 1N4, Canada; 9 Department of Pediatrics, University of Cincinnati College of Medicine, Cincinnati, OH 45229, USA

**Keywords:** *Xenopus laevis*, Normal table, Amphibian development, FETAX, EAMA, AMA, Metamorphosis, Embryo

## Abstract

Normal tables of development are essential for studies of embryogenesis, serving as an important resource for model organisms, including the frog *Xenopus laevis*. *Xenopus* has long been used to study developmental and cell biology, and is an increasingly important model for human birth defects and disease, genomics, proteomics and toxicology. Scientists utilize Nieuwkoop and Faber's classic ‘Normal Table of *Xenopus laevis* (Daudin)’ and accompanying illustrations to enable experimental reproducibility and reuse the illustrations in new publications and teaching. However, it is no longer possible to obtain permission for these copyrighted illustrations. We present 133 new, high-quality illustrations of *X. laevis* development from fertilization to metamorphosis, with additional views that were not available in the original collection. All the images are available on Xenbase, the *Xenopus* knowledgebase (http://www.xenbase.org/entry/zahn.do), for download and reuse under an attributable, non-commercial creative commons license. Additionally, we have compiled a ‘Landmarks Table’ of key morphological features and marker gene expression that can be used to distinguish stages quickly and reliably (https://www.xenbase.org/entry/landmarks-table.do). This new open-access resource will facilitate *Xenopus* research and teaching in the decades to come.

## Abstract (precis)

**Summary:** A new open-access Normal Table of *Xenopus* development is presented that integrates new embryo images with gene expression markers in Xenbase, the *Xenopus* biomedical research knowledgebase.

## INTRODUCTION

Normal tables of development are essential to allow different researchers to compare their results and to make experimental replication possible. Because the speed of *Xenopus* development depends upon the temperature of the water in which they grow, and these temperatures vary throughout the day and between individual laboratories, standardizing experiments to allow comparisons between laboratories and between experiments is inherently problematic. Since 1956, the standard reference for *Xenopus laevis* development has been the Normal Table of *Xenopus laevis* (Daudin) (hereafter referred to as the Normal Table), edited by Nieuwkoop and Faber (1956).

Chronicling the internal and external development of *Xenopus* ‘from the fertilized egg till the end of metamorphosis’, as the subtitle of the Nieuwkoop and Faber Normal Table states, started with field work to collect *X. laevis* from ponds near Stellenbosch in South Africa. The resulting work was an international collaboration of 28 embryologists, zoologists and field biologists, including the editors, from nine countries, all experts in the different organ systems in Anurans and other vertebrates. The primary data in the Normal Table was included in Chapter VI, expanded into 21 ‘divisions’, which detailed the temporal development of organs and systems including the nervous systems and sense organs; the early axial system; the skeleton and musculature of head, trunk and tail; the lateral line, skin and pigmentation; brain; cephalic nerves; spine and spinal ganglia; eye; head; heart and vasculature; gonads, adrenal glands; and the intestinal tract. The last two divisions (XX and XXI) focused on the gross anatomical and histological changes in the alimentary system, such as intestinal gut coiling. Chapter VII then summarized all external and internal criteria in detail for each developmental stage in chronological order from Nieuwkoop and Faber (NF) stage 1 (fertilized egg) to NF stage 66 (the froglet) (Nieuwkoop and Faber, 1956, 1994). Internal development was elucidated by histological analysis, and although this data was extensively referenced, it was not included in the Normal Table (for a complete historical overview, see Hopwood, 2007). A later publication, an atlas of the histology of early *Xenopus* development by Hausen and Riebesell in 1991 addressed the need for an authoritative histological reference (Hausen and Riebesell, 1991).

Nieuwkoop and Faber based their staging system on discrete external morphology and internal features at a stable temperature, rather than hours post-fertilization or length of larvae, as had been done for earlier vertebrate normal tables. Thus, the NF staging system can be applied to many other *Xenopus* species, including *X. tropicalis*, a physically smaller species widely used in disease modeling owing to its simpler diploid genome (*X. laevis* is pseudotetraploid) (Khokha et al., 2002), and even the exceptionally large, dodecaploid *X. longipes*, an endangered species being reared in captivity in which tadpoles metamorphose at near full adult body size (Tapley et al., 2015). Although the length of tadpoles at each NF stage varies amongst species, the internal and external milestones, such as early cell divisions (NF stage 2-6), the beginning of gastrulation (NF stage 10), the beating of the heart (NF stage 33 and 34), gut coiling (NF stage 41-46) or limb development (NF stage 48-58) can be used to stage embryos in most *Xenopus* species.

One of the most frequently used parts of the Normal Table is the set of 125 drawings by J. J. Prijs. Created in the style of embryologists of the era, these were based on pencil drawings Job Faber made on his South African field trip. These illustrations were initially used to great advantage by the Nieuwkoop laboratory during the pre-genomic era to characterize the timing of inductive signaling involved in the formation of the mesoderm germ layer and the neural ectoderm (Nieuwkoop, 1973, 1985; Durston et al., 1989). Others quickly appreciated the advantages of precise staging to characterize when and where critical developmental events occurred, from germ layer induction to organ formation. From the start of molecular embryology in the 1990s, to the current single-cell genomic era, accurate developmental staging remains a crucial step in any experiment.

Now, more than 65 years after the original publication, J. J. Prijs' classic drawings are considered an essential resource and are used extensively in teaching and research. There has always been a demand to reuse these drawings in publications, yet it is no longer possible to obtain copyright permission. Nieuwkoop and Faber personally held copyright as editors, and this was reinstated in subsequent reprintings in 1967, 1975, and lastly in 1994. Xenbase was given permission to display the entire series NF drawings in 2008, and these can be downloaded for personal use; however, to the best of our knowledge, the Nieuwkoop and Faber images are not in the public domain and still require permission for subsequent reuse in publications. Herein lies the conundrum: it is no longer possible to obtain re-use permission as the copyright holders are both deceased, and the publisher of the latest edition, Garland Publishing, Inc. (New York and London), is out of business. There is literally no one from whom to request permission.

Aside from the copyright issues, a few limitations of the Normal Table drawings have become apparent over the years. A number of important views of embryos and tadpoles are missing from the collection. For example, there are no anterior views after NF stage 21, no ventral views of neurula or early tadpoles at NF stages 13-40, dorsal views from NF stages 28-59 are not included, and views of larger tadpoles and metamorphic stages are truncated, omitting the tail. In addition, the gut-coiling diagrams that accompanied NF stage 43-45 lack sufficient detail to reflect accurately stage-specific changes in digestive system development. To address these limitations, we generated a new, open-source graphical resource to be used in conjunction with the original Normal Table text. This new resource includes 133 new high-quality illustrations of *X. laevis* development, from the fertilized egg to metamorphosing tadpoles and froglets, adding to a set of 30 previously released Zahn drawings that focused on anterior views of craniofacial development from the XenHead project (Zahn et al., 2017). Importantly, the Zahn drawings have a total of 67 new views that were not in the original Normal Table, new gut-coiling diagrams and new images of metamorphic stages. Finally, we have compiled an extensive reference table that summarizes key morphological landmarks and gene expression markers that can be used to distinguish quickly and reliably one stage from the next for NF stages 1-66 (Table S1).

All the images in the Zahn series are available on Xenbase, the *Xenopus* knowledgebase (http://www.xenbase.org/entry/zahn.do), and can be downloaded and reused under an attributable, non-commercial creative commons license. This new open-access resource will enable *Xenopus* research and teaching in the decades to come.

## RESOURCE DESCRIPTION

### Planning the illustrations

We first compared the existing 125 Nieuwkoop and Faber illustrations, and the 30 Zahn drawings from the XenHead project (Zahn et al., 2017), to all potential views of *Xenopus* embryonic stages (NF stages 1-66) (Fig. S1). To define a scope of work we prioritized: (1) standard views (dorsal, lateral, ventral, anterior); (2) stages most frequently assessed in *Xenopus* research (based on curation of *Xenopus* literature in Xenbase); while (3) attempting to cover comprehensively development from fertilization to metamorphosis; and (4) adding additional views not included in the 1956 Normal Table, but deemed useful to current *Xenopus* research (e.g. anterior and ventral views). The result was a scope of work with 133 illustrations of 56 developmental stages, spanning fertilized egg to froglet stages, including a composite illustration of hindlimb and forelimb development and new gut-coiling diagrams. Of these drawings, 100 are fully rendered and shaded, and 33 are unshaded line drawings. In total, the Zahn collection now consists of 198 images of *X. laevis*, covering 60 of the 66 NF developmental stages, 73 more than the original 1956 artwork by J. J. Prijs (Fig. S1).

### *Xenopus laevis* staging landmarks

Staging *Xenopus* embryos is a skill that takes considerable practice to master. Our concept for a series of ‘staging landmarks’ arose from students’ comments highlighting the need for a simplified ‘staging for beginners’ chart. Our goal was to generate a list of external anatomical features that experienced bench scientists look for when staging embryos, which could be used by students at the bench and by newcomers to *Xenopus* development, in addition to seasoned experts. We liken the concept of *Xenopus* staging landmarks to ‘birding field marks’, a simple yet distinctive combination of just a few traits that birders use to identify morphologically similar bird species in the field (e.g. great egrets have a yellow bill and black feet, whereas snowy egrets have a black bill and yellow feet). With input from experienced *Xenopus* researchers, and with careful reference to descriptions from the 1994 edition of the Normal Table (Nieuwkoop and Faber, 1994), we distilled the ‘external’ morphological traits that distinguish each stage down to just a few key attributes that are visible under a stereomicroscope. Internal landmarks include major developmental processes and milestones from the Normal Table and findings from recent published research.

Leveraging decades of *in situ* hybridization (ISH) and immunohistochemistry (IHC) data curated in Xenbase, the Landmarks Table includes key gene expression markers of specific tissues and organs, data that was unknown at the time of the original Normal Table's publication, but which are now standard tools in most research laboratories. Importantly, molecular markers often indicate tissues and organ primordia prior to the overt formation of anatomical structures. For example, expression of the homeobox gene *nkx2-1* can be detected by ISH in the respiratory epithelium as early as NF stage 33-34, prior to the emergence of lung buds at NF stage 40 (Rankin et al., 2015).

The *Xenopus* staging Landmarks Table (Table S1) will be maintained on Xenbase (https://www.xenbase.org/entry/landmarks-table.do) as a living document, updated over time with community input and new research findings. Extensive gene expression data are also searchable on Xenbase using either the Expression Search tool (http://www.xenbase.org/geneExpression/geneExpressionSearch.do?method=display) or the *Xenopus* Anatomy Ontology (XAO)/Search Anatomy menu (http://www.xenbase.org/anatomy/anatomy.do?method=display&tabId=2). Curated marker gene lists, based on published and community submitted gene expression images, are also provided for hundreds of XAO terms on Xenbase (James-Zorn et al., 2018).

### Illustrations of *Xenopus laevis* development

#### NF stages 1-6: zygote and cleavage

**Fig. 1.**
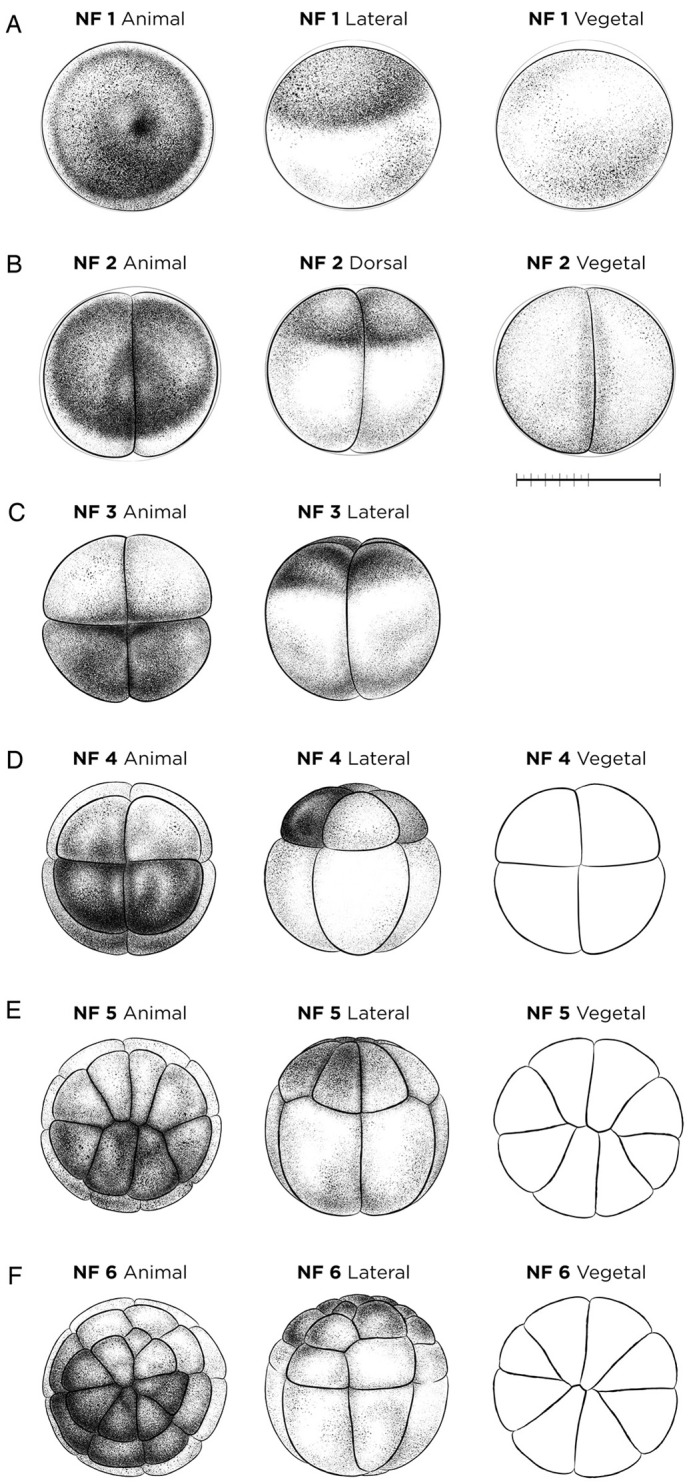
**Cleavage-stage *X. laevis* embryos.** (A) The fertilized egg NF stage 1. (B) NF stage 2 (two-cell stage). (C) NF stage 3 (four-cell stage). (D) NF stage 4 (eight-cell stage). (E) NF stage 5 (16-cell stage). (F) NF stage 6 (32-cell stage). Vegetal/ventral views of NF stage 4-6 (D-F) are unshaded line drawings. See Table S1 for staging landmarks. Views as indicated. Scale bar: 1 mm.

Approximately 20 min after fertilization, the *Xenopus* egg, NF stage 1, rotates within the vitelline membrane such that the darkly pigmented animal hemisphere faces up and a lighter vegetal hemisphere faces down (Sive et al., 2007b). A condensation of dark pigment in the animal cortex indicates the sperm entry point, and a pale spot is often visible where the germinal vesicle has broken down (Fig. 1A). At room temperature (23°C), the first cell division occurs approximately 90 min post-fertilization, dividing the embryo into right and left halves. The fully cleaved two-cell embryo is NF stage 2 (Fig. 1B). The second cleavage ∼20-30 min later divides the embryo into dorsal and ventral halves to produce a four-cell embryo, NF stage 3 (Fig. 1C). During the next four cell cycles, the embryo continues to undergo synchronous, holoblastic cell division every 20-30 min at 23°C. The third cell division plane is perpendicular to the second, dividing the embryo into animal and vegetal halves resulting in an eight-cell embryo, NF stage 4 (Fig. 1D). Two subsequent cell divisions generate NF stage 5 (16-cell) (Fig. 1E) and NF stage 6 (32-cell) embryos with four rows of eight cells (Fig. 1F). Animal and lateral views of NF stage 3 to NF stage 6 embryos illustrate the often-noticeable difference between the lighter dorsal-animal blastomeres and the darker ventral-animal blastomeres. In addition, the animal hemisphere cells are noticeably smaller than cells in the vegetal hemispheres (Fig. 1D-F). Early molecular makers for NF stage 2 include *atp4a*, which marks the animal hemisphere; *vegt*, which marks the vegetal hemisphere and *nanos1*, which marks the germ plasm at NF stage 4 (Betley et al., 2002; Stennard et al., 1996; Walentek et al., 2012).

#### NF stages 6.5-12.5: blastula, gastrula and early neurula

**Fig. 2.**
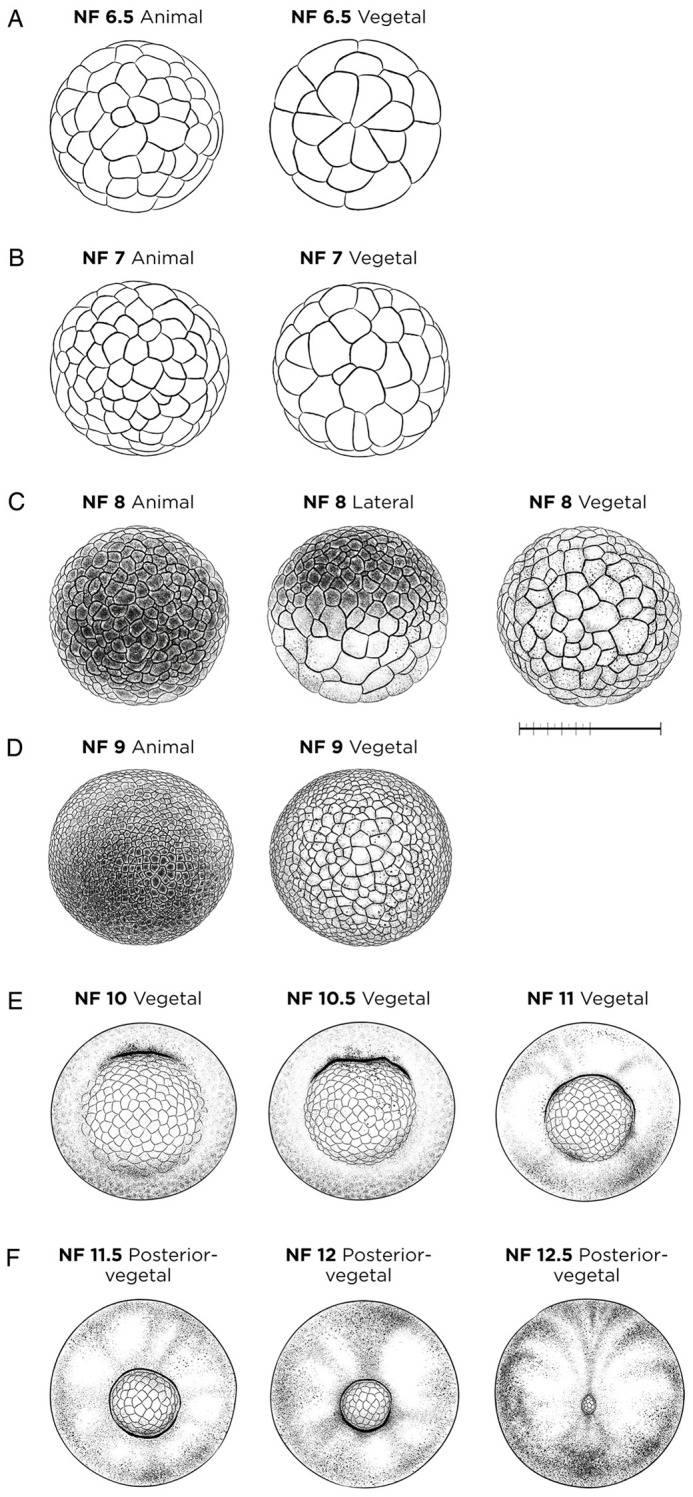
**Blastula-, gastrula- and early neurula-stage *X. laevis* embryos.** (A) NF stage 6.5 (morula); unshaded line drawing. (B) NF stage 7 (large-cell blastula); unshaded line drawing. (C) NF stage 8 (medium-cell blastula); membrane removed. (D) NF stage 9 (fine-cell blastula); membrane removed. (E) Gastrula-stage embryos, NF stage 10, NF stage 10.5 and NF stage 11; membrane removed. (F) NF stage 11.5, NF stage 12 and NF stage 12.5; membrane removed. See Table S1 for staging landmarks. Views as indicated. Scale bar: 1 mm.

Early *Xenopus* embryos undergo cell division without growing in size. By morula stage, NF stage 6.5 (Fig. 2A), cell division becomes asynchronous, being faster in the animal/dorsal than the vegetal/ventral region. At NF stage 7 (Fig. 2B), the fluid-filled blastopore cavity forms internally in the animal half of the embryo. It is no longer possible to count cell numbers reliably for NF stages 6.5-9, so the relative size of the darkly pigmented animal cells becomes the key feature distinguishing NF stage 8 (mid-blastula) (Fig. 2C) from NF stage 9 (late blastula) (Fig. 2D). This is an important staging watershed as early NF stage 8 marks the mid-blastula transition when, after 12 cell divisions, the cell cycle slows and large-scale zygotic transcription commences (Newport and Kirschner, 1982). At NF stage 7, *nodal5* is a molecular marker for the earliest evidence of zygotic transcription, and *gs17* and *nr1* are markers for the major initiation of zygotic transcription at NF stage 8. At late blastula stage, NF stage 9, *sox17* is a widely used marker for endoderm and *tbxt* (formerly known as *t* or *brachyury*) is the standard marker for mesoderm.

Gastrulation begins at NF stage 10 with the appearance of the blastopore lip in the vegetal dorsal region of the embryo (Fig. 2E). The invaginating dorsal blastopore lip becomes obvious as a darker indentation, caused by apical constriction of bottle cells and condensation of pigment (Moosmann et al., 2013). As gastrulation proceeds, the involuting marginal tissue at the blastopore lip extends laterally towards the ventral sides of the embryo, reaching almost halfway around the blastopore circumference at NF stage 10.5 (Fig. 2E). Between NF stages 11 and 12 (Fig. 2E,F) the vegetal cells are increasingly internalized, and the blastopore diameter progressively decreases, owing in large part to mediolateral intercalation of the underlying mesodermal cells (Keller and Sutherland, 2020). The relative dimensions of yolk plug to blastopore diameter, in combination with the extent of pigmentation along the blastopore lip, are key landmarks for stage determination during gastrulation (Fig. 2E,F). Based on molecular analysis, it is now clear that neural induction begins as early as NF stage 10.5 with *sox2* expression in the dorsal ectoderm (Gawantka et al., 1998). The neural plate is identifiable by NF stage 12 (Fig. 2F) with a posterior constriction of the midline dorsal ectoderm, and by NF stage 12.5 (blastopore closed completely) (Fig. 2F) the notochord forms from mediolateral convergence of dorsal axial mesoderm under the neural ectoderm, a process identifiable by *chrd.1*, *nog* and *shh* expression (Roelink et al., 1994; Sasai, 1994; Fletcher and Harland, 2008).

#### NF stages 13-21: neurula

**Fig. 3.**
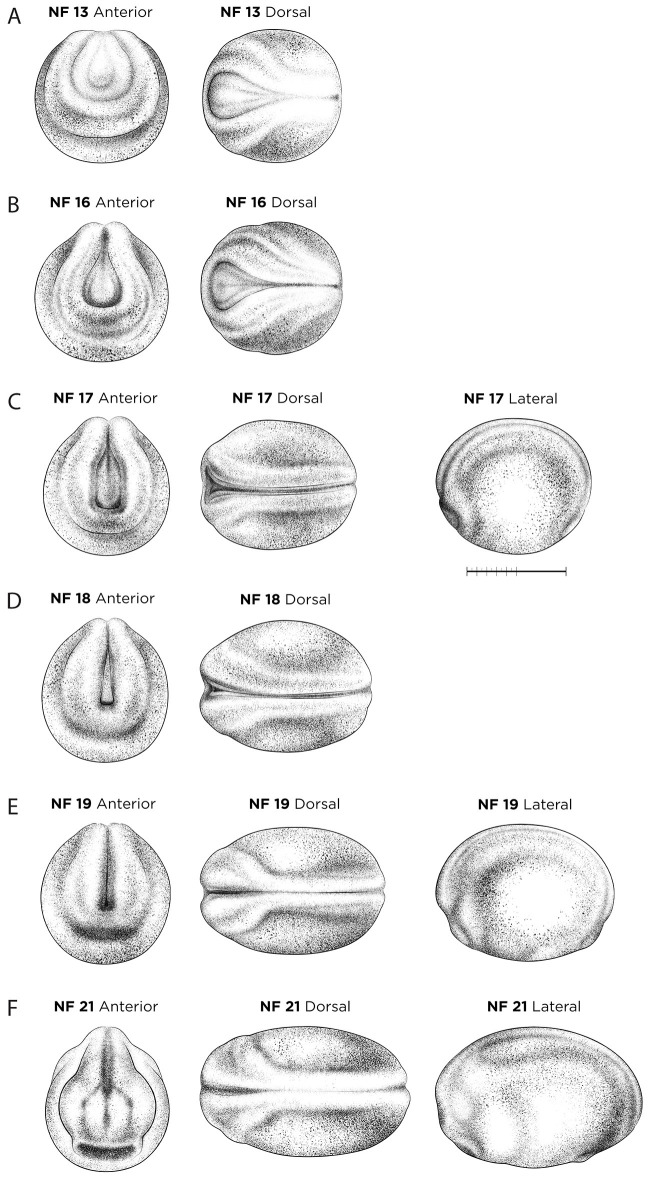
**Neural-stage *X. laevis* embryos.** (A) NF stage 13 (slit-blastopore). (B) NF stage 16 (mid-neural fold). (C) NF stage 17 (late neural fold). (D) NF stage 18 (neural groove). (E) NF stage 19 (initial neural tube). (F) NF stage 21 (suture of neural groove completely closed). Orientation for anterior views is dorsal up; dorsal views have anterior left; lateral views have dorsal up and anterior left. See Table S1 for staging landmarks. Membrane removed in all embryos. Views as indicated. Scale bar: 1 mm.

During neurulation, the neural plate deepens along the midline, and folds onto itself to form the neural tube (which later develops into the spinal cord and brain), a process which proceeds in a posterior to anterior direction. Accurate staging during early neurulation (Fig. 3A-E), NF stage 13 to NF stage 19 is very challenging as it is based on subtle differences in three traits: the three-dimensional shape of the neural folds, the width of the neural plate and the degree of neural tube closure. To visualize these features optimally, embryos are removed from the vitelline membrane (Sive et al., 2007a) so that the neural plate is not compressed. Internally, at NF stage 19 (Fig. 3E), the cranial neural crest forms at the border of the anterior neural plate and begins to migrate in ‘streams’ towards the ventral surface. A classic marker for early cranial neural crest streams is *sox9*, although many other genes also mark migrating neural crest cells (Spokony et al., 2002; Kuriyama and Mayor, 2008, 2009). NF stage 21 (Fig. 3F) is a popular stage to assay and staging is relatively easy, as the embryo is still curved dorsally, the neural tube is completely closed, and the developing eyes (optic vesicles) are visible as ‘oblique oval spots’ in lateral view and as slight bulges when viewed anteriorly. Molecular markers for NF stage 21 include *pax6* for the optic vesicle (Nakayama et al., 2015), *tekt3* and *foxji* for multiciliated epidermal cells on the epidermis (Song et al., 2014; Chung et al., 2014), and *pax8* and *lim1* for the pronephric mesenchyme (Vize et al., 1995).

#### NF stages 22-28: tailbud stages

**Fig. 4.**
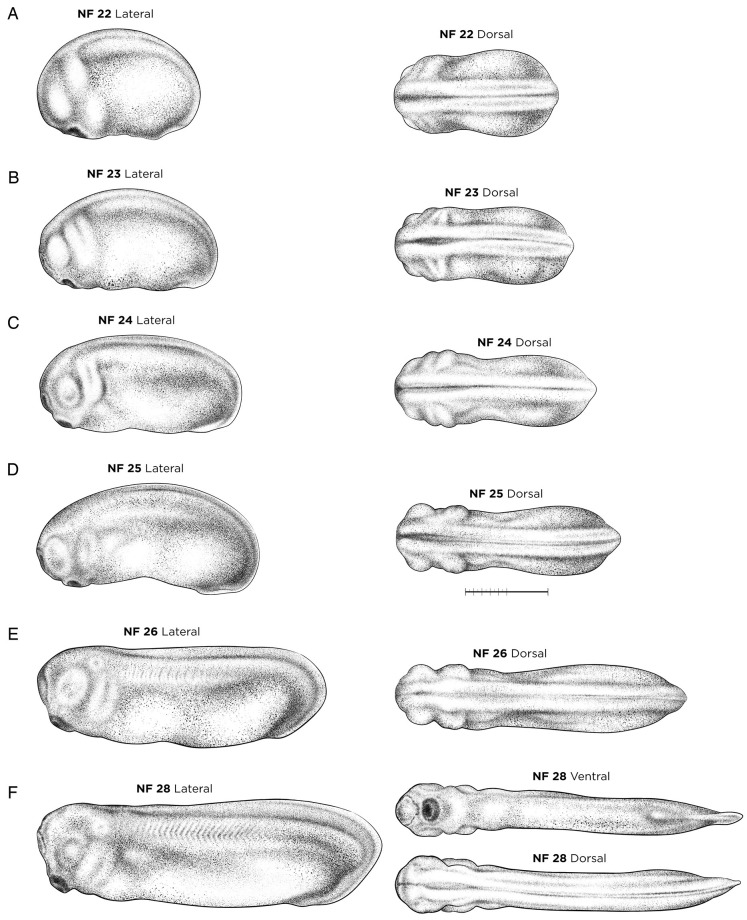
**Early tailbud-stage *X. laevis* embryos.** (A) NF stage 22. (B) NF stage 23. (C) NF stage 24. (D) NF stage 25. (E) NF stage 26. (F) NF stage 28. Orientation for dorsal and ventral views is anterior left; lateral views have dorsal up and anterior left. See Table S1 for staging landmarks. Membrane removed in all embryos. Views as indicated. Scale bar: 1 mm.

Anatomical landmarks for early tailbud embryos NF stages 22-26 (Fig. 4A-E) include the extent of dorsal curvature in the embryo, the number of pharyngeal ‘bulges’, the three-dimensionality and pigmentation of the developing eye, and the number/extent of anteriorly segregated, chevron-like somites. During this period, the somites, heart and pronephric kidney begin to form, while the embryo lengthens and becomes noticeably more slender. Although somite segmentation can be difficult to see under a light microscope until ∼NF stages 27-28, molecular markers such as *myod1* can clarify the boundary between the formed somites and the presomitic mesoderm from NF stages 22 (Fig. 4A) onwards (Hopwood et al., 1992; Shang et al., 2020). NF stage 23 (Fig. 4B), referred to as a ‘coffee bean’ embryo, develops the inverted Y-shape, hatching gland on the anterior ‘forehead’, which expresses *cxcl14*, *astl3a.1* and *pax3* (Park et al., 2009). Key neurobehavioral milestones for tailbud stages include motor reactions to external stimuli at NF stage 24 and spontaneous movements at NF stage 26 (Fig. 4E), and at NF stage 28 (Fig. 4F) embryos that have been liberated from their vitelline membrane glide around a Petri dish as a result of the ciliary beating across the epidermis.

External landmarks for NF stage 28 (Fig. 4F) include a fully pigmented cement gland, the fin with an outer transparent and an inner translucent band that now extends to the cloaca, and a characteristic ‘nose shape’ to the extending tailbud. Internal landmarks for NF stage 28 include four distinct streams of *dlx2*-positive migrating cranial neural crest cells (Square et al., 2015), segregation of the epibranchial placodes, which express *neurog2*, *foxi2* and *pax2* (Schlosser, 2006; Saint-Jeannet and Moody, 2014), and the first appearance of *pax2-* and *lhx1*-expressing nephrostomes (Vize et al., 1995; de Bakker et al., 2019). The heart of an NF stage 28 embryo has an endocardial tube surrounded by myocardium (marked by *tnni3*) and the beginning of the pericardial cavity. The otic vesicles (the auditory system primordia) detach from the epidermis at NF stage 28, yet are difficult to see under light microscopes, although they are commonly detected by *sox3*, *sox9* and *eya1* expression (Saint-Germain et al., 2004; Almasoudi and Schlosser, 2021). Anteriorly, *fgf8* and *frzb1* mark the mouth primordium at NF stage 28 (Kennedy and Dickinson, 2012), and the complete formation of the hypochord (ventral to the notochord) can be seen by ISH for *vegfa* and *rspo3* (Cleaver et al., 1997; Kazanskaya et al., 2008).

#### NF stages 29-38: late tailbud and free-swimming tadpoles

**Fig. 5.**
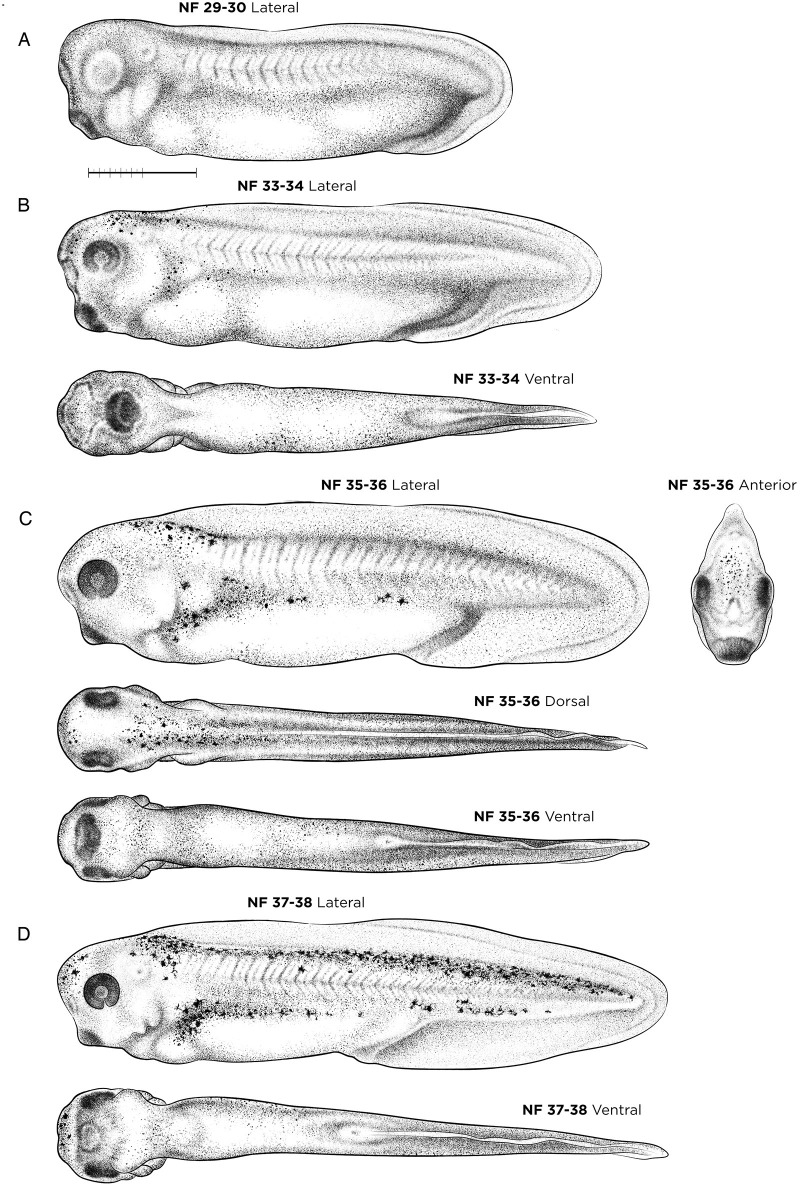
**Late tailbud-stage and free-swimming tadpole *X. laevis* embryos.** (A) NF stage 29-30. (B) NF stage 33-34. (C) NF stage 35-36. (D) NF stage 37-38. Orientation for dorsal and ventral views is anterior left; lateral views have dorsal up and anterior left. See Table S1 for staging landmarks. Membrane removed in all embryos. Views as indicated. Scale bar: 1 mm.

Staging of late tailbud stages is best accomplished after removal of the vitelline membrane, focusing on eye morphology, tail and gut development, and the spread of melanophores across the body (Table S1). The developing eye cup forms a gray disc at NF stage 29-30 (Fig. 5A) and its darkening color and degree of choroid fissure closure are easy to assess at NF stage 33-34 (darker above, still gray ventrally, open C-shape) (Fig. 5B), NF stage 35-36 (completely black, edges almost touching) (Fig. 5C) and NF stage 37-38 (fissure surfaces touching, but still slightly open) (Fig. 5D).

As embryos lengthen as a result of tail growth, their abdominal area simultaneously ‘shortens’ as undifferentiated gut endoderm begins to form distinct fore-, mid- and hindgut domains. The ‘tail length to gut length’ ratio is used as an external landmark (see Table S1), as is the angle formed between the tail and the posterior end of the gut (the future proctodeum) to distinguish NF stage 37-38 (140°), NF stage 39 (125°) and NF stage 40 (90°). Additional external landmarks include the extent of migration of the pigmented melanophores over the head and trunk starting at NF stage 33, and along the tail by NF stage 39. NF 35-36 marks a major behavioral transition as tadpoles naturally break free from their vitelline membrane to become free-swimming larvae/tadpoles.

A number of internal organogenesis milestones occur between NF stages 29 and 38 (Fig. 5). The embryonic heart forms as a linear tube with an anterior outflow tract, left ventricle, atrioventricular canal and atrium by NF stage 32. Heart looping and spontaneous heart contractions begin at NF stage 33-34 (Fig. 5B), with the heart obtaining a distinct S-shape with a separate atrium and ventricle by NF stage 35-36 (Fig. 5C). *Xenopus* heart development can be visualized by ISH with the following markers: *hand2*, *tnni3* and *actc1* for the early heart (endocardial tube and cardiac mesoderm), and *aplnr* for blood vessels (Drysdale et al., 1994; Parain et al., 2012; Vokes and Krieg, 2002). Between NF stage 32 and 35, asymmetric expression of *pitx2* and *bmp4* is detected in the looping heart tube (Breckenridge et al., 2001). Blood flowing in the vasculature is visible by NF stage 37-38 (Fig. 5D), and *hba3* marks the ventral blood island (Kelley et al., 1994).

During this period, the bilateral pronephric kidney begins filtering the blood and osmoregulation. The glomus (where blood is filtered), nephrostomes (which collect the urine/filtrate) and pronephric duct (which drains the filtrate) form from the nephrogenic intermediate mesoderm by NF stage 28-30 (Beckers et al., 2021; Raciti et al., 2008; Zhou and Vize, 2004). The pronephric duct fuses with the rectal diverticulum at NF stage 35-36, such that the entire pronephric kidney is functional by NF stage 37-38. The glomus expresses *wt1*, *nph1* and *cndp*, whereas the nephrostomes express *cfap161*, *pax2* and *lhx1* (Li et al., 2012; Beckers et al., 2021). Once epithelialized, the entire pronephric kidney and pronephric duct express *atp1a* and *slc4a4* along with various segment-specific solute transporters (Vize et al., 1995; de Bakker et al., 2019).

During NF stages 29-38 significant patterning also occurs in the central nervous tissue and digestive system. For example, *en2* (also known as *krox20*), *pax2* and *fgf8* mark the midbrain-hindbrain boundary (Brivanlou and Harland, 1989; Heller and Brändli, 1997; Lea et al., 2009), whereas *gsx1* identifies distinct regions of the forebrain (e.g. hypothalamus, zona limitans intrathalmica, medial ganglionic eminence and pretecum) and the hindbrain rhombomeres (r1-r5, r7 and r8) at NF stage 31-32 (Illes et al., 2009). In the gut tube, *hhex* marks the liver bud and *ptf1a* and *pdia2* the pancreatic buds (Pan et al., 2007; Zorn and Mason, 2001), whereas *nkx2-1* marks the thyroid gland and lung buds at NF stage 33-34 (Rankin et al., 2015).

#### NF stages 40-46: free-swimming tadpole

**Fig. 6.**
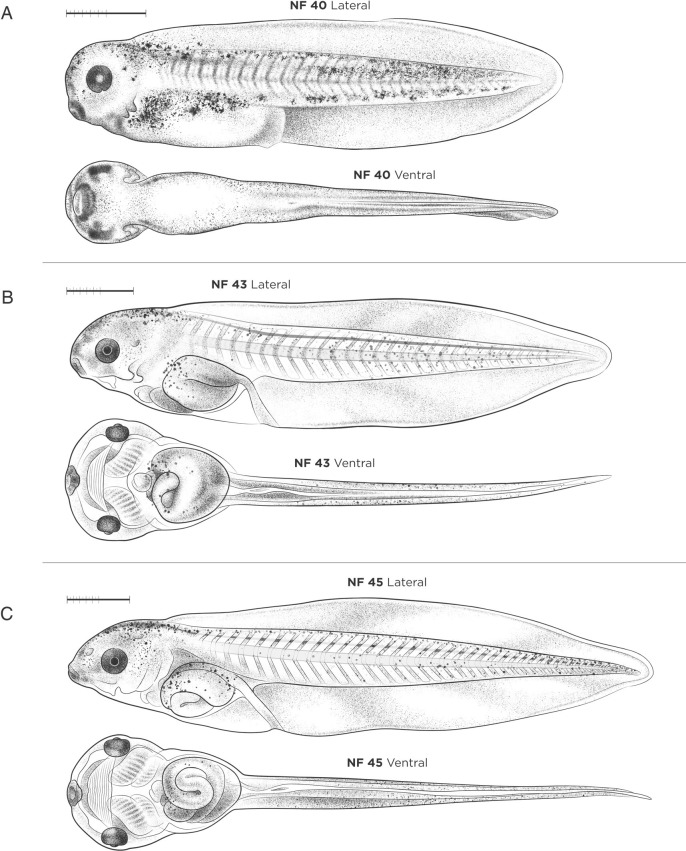
**Free-swimming and gut-coiling stages of *X. laevis* tadpoles.** (A) NF stage 40. (B) NF stage 43. (C) NF stage 45. Orientation for ventral views is anterior left; lateral views have dorsal up and anterior left. See Table S1 for staging landmarks. Membrane removed in all embryos. Views as indicated. Scale bars: 1 mm.

**Fig. 7.**
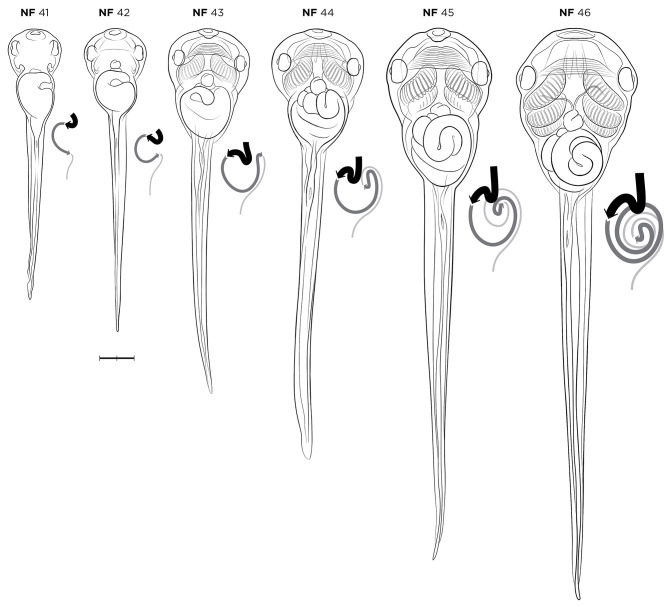
***X. laevis* embryos during gut-coiling stages, NF stages 41-46, in ventral view, alongside new gut coiling diagrams.** The coiling digestive tract is depicted as three lines of varying thickness. The esophagus/stomach (thickest black line) begins anteriorly on the left side of the body at NF stage 41. As gut lengthening and coiling progresses, the stomach shifts to the right side of the body by NF stage 46. The midgut (dark-gray line) and hindgut (thin light-gray line) form a rudimentary ‘S’ shape curve by NF stage 41-42, and at NF stage 43 the midgut and hindgut have lengthened to form a ‘hairpin loop’, visible from the left side. This loop turns ventrally by NF stage 44, becoming the U-shaped apex of the future intestinal coils. Throughout NF stages 44 to 46, the midgut and hindgut continue to lengthen and loop, with the apex rotating inward to form a compact intestine with tightly wound, counterclockwise coils. Gut-coiling diagrams designed by J.G. Stages as indicated. Scale bar: 1 mm.

Morphogenesis of the digestive and respiratory systems are the major milestones occurring during the late free-swimming tadpole and pre-metamorphosis stages (Figs 6A-C and 7). Many of these events can be readily observed as the body wall of tadpoles becomes more transparent. The foregut gives rise to the esophagus, trachea, lungs, stomach, duodenum, liver, pancreas and gall bladder, whereas the midgut and hindgut give rise to the intestine and cloaca, respectively. At NF stage 40 (Fig. 6A), external landmarks include the completely closed choroidal fissure and clearly visible blood circulating through the gills. NF stage 40 tadpoles will take gulps air from the surface at this stage, although their lungs are not yet functional. Internally, the liver and pancreatic organ buds are obvious at NF stage 41 as the intestine begins its first leftward curvature (Fig. 7). The tissue occluding the mouth begins to break down between NF stages 40 and 41, and at NF stage 42 the common foregut tube is completely divided with the trachea and lungs separated from the esophagus (Nasr et al., 2019). Tadpoles start to feed at NF stage 45 (Fig. 6C) and food can be seen in the gut. As gut development and elongation proceeds through NF stages 43-47 (Figs 6B,C and 7), stereotypical gut coiling and asymmetry of the viscera/organs is best viewed ventrally.

New drawings of stages NF 41-46 in ventral view, alongside the new gut-coiling diagrams are shown in Fig. 7. The coiling digestive tract is depicted as three shaded lines of varying thickness, representing the foregut, midgut and hindgut. The esophagus/stomach (thick, black line) begins on the left side of the body at NF stage 41, lengthening extensively to situate the stomach on the right side of the body by NF stage 46. By NF stages 41-42, the midgut (dark-gray line) and hindgut (thin, light-gray line) form a rudimentary S-shaped curve. By NF stage 43, the midgut and hindgut have lengthened to form a ‘hairpin loop’ visible from the left side. This loop turns ventrally by NF stage 44, and will become the U-shaped apex (i.e. center) of the future intestinal coils. Between NF stages 44 and 46, the midgut and hindgut continue to lengthen, with the apex rotating inward to form an intestine with tightly wound, counter-clockwise coils.

Other landmarks at NF stages 44-46 involve skeletal growth, neural development and the visual system. Retinal ganglion cells reach the optic tectum and begin to form complex synapses in a stage-specific manner during NF stages 44-46 (Pratt, 2021). Visual avoidance behavior, whereby tadpoles swim away from dark spots/shadows, is first observed at NF stage 44 (Dong et al., 2009). At the end of the gut-coiling stages, the hindlimbs develop first, emerging as tiny crescent-shaped buds at NF stage 46 (although they can be difficult to see) and chondrification of the otic vesicle and parts of the skeleton begin to develop as the notochord starts to degenerate.

#### NF stages 47-61: premetamorphosis and prometamorphosis

**Fig. 8.**
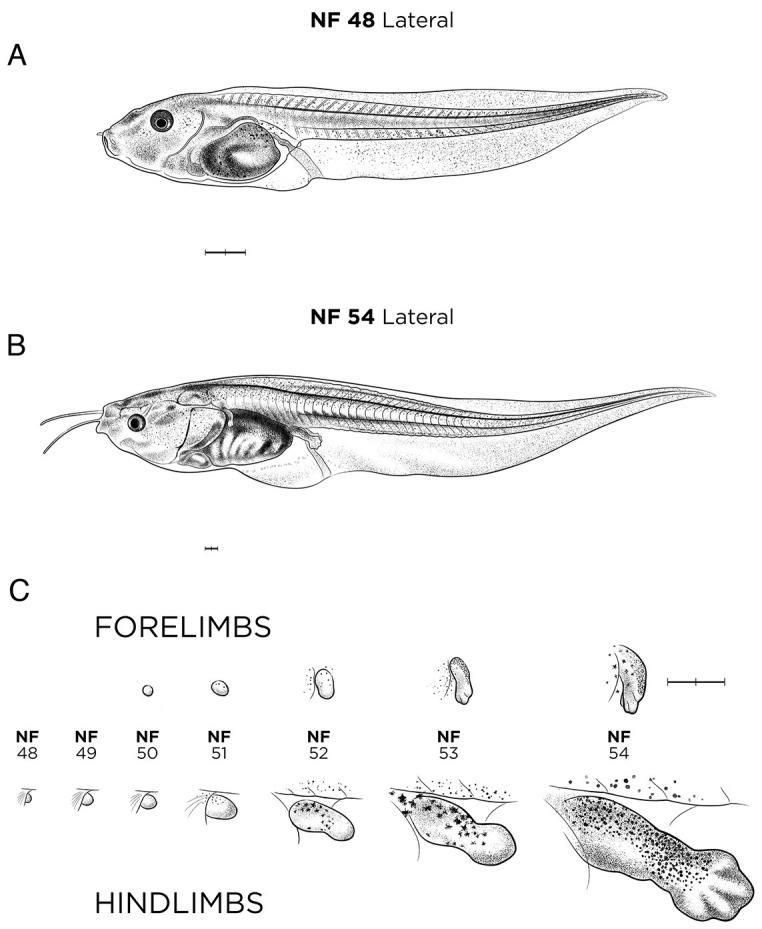
**Limb development in *X. laevis* tadpoles.** (A) NF stage 48 tadpole. (B) NF stage 54. (C) Limb bud development from NF stage 48 to 54, reproduced in the style of the drawing in the Nieuwkoop and Faber Normal Table (Nieuwkoop and Faber, 1956, 1994), but in left-to-right progression, with forelimbs (above) and hindlimbs (below), at the stages indicated. See Table S1 for staging landmarks. Views as indicated. Scale bars: 1 mm.

**Fig. 9.**
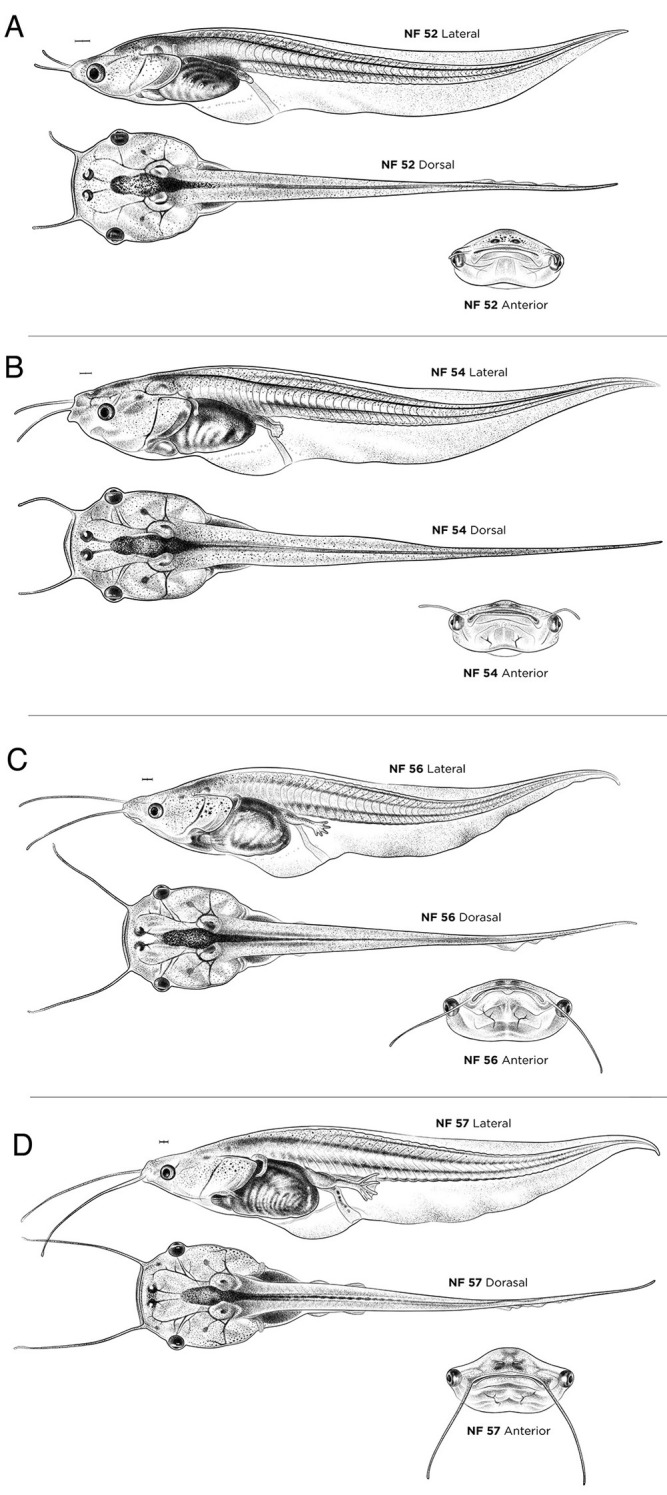
**Premetamorphosis- and prometamorphosis-stage *X. laevis* tadpoles.** (A) NF stage 52 (premetamorphosis). (B) NF stage 54 (premetamorphosis). (C) NF stage 56 (prometamorphosis). (D) NF stage 57, (prometamorphosis). Each stage is shown in lateral, dorsal and anterior views. Ventral views are available on Xenbase. See Table S1 for more staging landmarks. Membrane removed in all embryos. Views as indicated. Scale bars: 1 mm.

**Fig. 10.**
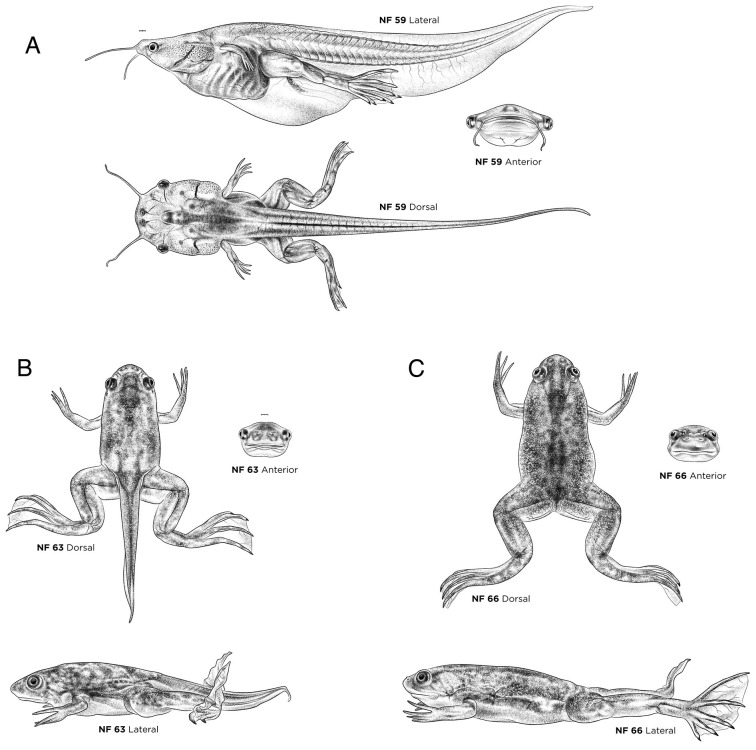
**Prometamorphosis- and climax metamorphosis-stage *X. laevis* tadpoles.** (A) NF stage 59. (B) NF stage 63. (C) NF stage 66. Each stage is shown in lateral, dorsal and anterior views. Ventral views are available on Xenbase. See Table S1 for more staging landmarks. Membrane removed in all embryos. Views as indicated. Scale bars: 1 mm.

Amphibian metamorphosis involves numerous, precisely timed developmental changes, including intestinal remodeling, limb growth, vascular reorganization, changes to the neuronal circuitry, tail resorption, simultaneous gill resorption and lung maturation, and the change from ciliated larval epidermis to adult skin (Brown and Cai, 2007; Choi et al., 2017; Tasca et al., 2021). The three phases of metamorphosis (premetamorphosis, prometamorphosis and climax metamorphosis) are represented by NF stages 48-59 (Figs 8A,B, 9 and 10A), NF stage 63 (tailed froglet; Fig. 10B) and NF stage 66 (froglet with a fully resorbed tail; Fig. 10C). Additional ventral views of these stages are available on Xenbase.

Landmarks for premetamorphosis (NF stages 47-54) focus on limb growth and increasing numbers of pigmented melanophores (Table S1). At NF stage 47, the ‘atria’, where forelimb will develop, is formed; however, forelimb buds are not visible until NF stage 50 (Fig. 8). At NF stage 52 (Fig. 9A), the hindlimb has an indented wrist, and by NF stage 54 (Fig. 9B) the foot forms as a splayed paddle with five thickened digits and thinner inter-digital webbing. At the molecular level, *fgf8*, *fgf2* and *sall4* are widely used markers for hindlimb bud, and *fgf2* for the forelimb bud. Melanophores form an opaque layer surrounding the abdominal cavity and the intestinal coils at NF stage 47, and these become iridescent gold by NF stage 48. Additional landmarks for these stages include the developing brain, where the axon tracts forming between the retinal ganglionic cells and the optic tectum develop in a stage-specific progression (Liu et al., 2016). Furthermore, tadpoles at late premetamorphosis stages are regeneration competent, in that full regeneration of a lost tail or limbs is possible (Aztekin et al., 2021).

During prometamorphosis (NF stages 55-61), the respiratory system changes from gills to lungs and the limb musculature and skeleton mature. The relative proportions of limb segments and the position of the growing limbs are used as key staging landmarks. At NF stage 55 (not shown), the forelimb hand rotates 90° and the digits/fingers are as long as they are wide. At NF stage 56 (Fig. 9C), the hindlimbs start to be visible from above because they can rotate away from body. At NF stage 57 (Fig. 9D), the forelimb is still covered by the operculum membrane. Tadpoles at NF stage 55-56 are considered ‘regeneration restricted’ in that complete regeneration of a limb is no longer possible, and at NF stage 58 onwards tadpoles are completely ‘regeneration incompetent’ (Aztekin et al., 2021).

Levels of the thyroid hormones (TH) thyroxine (T_4_) and 3,5,3′-l-triiodothyronine (T_3_) are the key driver of metamorphosis (Table S1). First detectable at NF stage 47, TH levels dramatically increase from NF stage 54 (Fig. 8B) to NF stages 55-61, peaking at NF stage 62-63 (Fig. 10B), and returning to prometamorphic levels by NF stage 66 (Fig. 10C) (Buchholz and Shi, 2018).

#### NF stages 58-66: climax of metamorphosis

Climax of metamorphosis, when tail and the gills are resorbed, culminates in an air-breathing, yet still fully aquatic, *Xenopus* froglet. At NF stage 58, ∼44 days after fertilization at 23°C, the forelimbs erupt, usually elbows first (although left and right forelimbs may not emerge simultaneously), and the claws form on the tips of hindlimb digit tips (they are white at first). At NF stage 59 (Fig. 10A), the hindlimb claws (from which the species gets its common name) turn hard and black, the shortest toes first, and the sensory barbels begin to shrivel up. A common molecular marker for the cartilage elements of developing digits is *sox9*, whereas *tbx4* and *sall4* mark the interdigital mesenchyme (Neff et al., 2005). By NF stage 61, both hindlimb and forelimb are fully formed, the adult skin has developed across the body, and tadpoles cease feeding owing to oral and intestinal remodeling (Choi et al., 2017). Significant changes in the shape of the head, relative position of the mouth and eyes, and the natural positions of the limbs can be observed by comparing NF stage 63 (Fig. 10B) and NF stage 66 (Fig. 10C), many of which are useful staging landmarks.

## DISCUSSION

We have created a new open-source set of illustrations showing the Normal Table of *Xenopus* development from fertilization through embryonic development, to fully metamorphosized froglet, to be used in conjunction with text of the classic Nieuwkoop and Faber Normal Table (Nieuwkoop and Faber, 1994) and the histology atlas of Hausen and Riebesell (1991). This new online digital resource, with images in the classic Nieuwkoop and Faber style, will overcome the current limitations in reusing the original illustrations, whereby it is impossible to obtain reuse copyright permission. The new Zahn collection also includes additional views that were not presented in the original Normal Table, but are valuable for current research. We also include line diagrams of some key stages, such as cleavage and blastula, which are frequently modified and reused to illustrate experimental design in scientific papers.

Continuous morphological change is a natural feature of development, and, in practice, applying staging criterion is challenging, even for experts. When designing experiments, researchers can either wait until an easy-to-determine stage is reached, or halt experiments at a set time point and then ‘stage’ the embryos. Our Landmarks Table (Table S1) provides a succinct guide that can be used by experts and the inexperienced alike, to stage embryos quickly and reliably. The Landmarks Table not only includes external morphology, but also internal anatomical landmarks based on descriptions in the Nieuwkoop and Faber Normal Table (Nieuwkoop and Faber, 1994) and the histological images in Hausen and Riebesell (1991), with input from the *Xenopus* community. Finally, the stage- and tissue-specific molecular markers were curated from decades of *Xenopus* research data in Xenbase.

The experimental advantages of *Xenopus* include a well-defined fate map, targeted microinjection, experimental embryology, rapid CRISPR-gene editing, transgenics and older tadpoles that are largely transparent making it easy to observe organogenesis in real time. As a result, *X. laevis* and *X. tropicalis* are used to model an increasing variety of human diseases affecting various organ systems as well as to elucidate basic principles of cell biology, genomics and morphogenesis, while informing advances in stem cell and organoid technology, injury, repair and regeneration, and toxicology (Sater and Moody, 2017; Nenni et al., 2019; Hoppler and Conlon, 2020; Gao and Shen, 2021). All disease modeling studies rely on accurate, reproducible staging and an understanding of a normal wild-type *Xenopus* phenotype. The Zahn drawing series provides a realistic, highly detailed study of anatomically ‘normal’ embryos and tadpoles, by which ‘abnormal’ phenotypes can be assessed. The new anterior views of tadpoles, for example, may be a particularly useful reference to assess craniofacial defects, for which it is important to assess the relative positioning of the mouth, nose and eyes (Wyatt et al., 2021b).

The new ventral views of *Xenopus* tadpoles from NF stages 41-46 and diagrams of gut coiling will enable researchers to model more accurately congenital defects of the digestive system and visceral organs, such as situs inversus, heterotaxia, straight or shortened gut, reversed intestinal coiling or malrotation (Bergmann et al., 2008; Gur et al., 2017; Dush and Nascone-Yoder, 2019; Wyatt et al., 2021a). The gut-coiling diagrams will also facilitate the use of *Xenopus* for toxicology research, as digestive tract phenotypes are a common outcome of the ‘Frog Embryo Teratogenesis Assay *Xenopus*’ (FETAX), an industry standard used to assess the developmental toxicity of pharmaceuticals and food supplements (Islas-Flores et al., 2018; Fort and Mathis, 2018; Battistoni et al., 2020), agricultural chemicals (Babalola et al., 2021) and other pollutants (Mouche et al., 2017; Xu et al., 2019). FETAX phenotypes are generally assessed by whole-mount microscopy of late tadpole stages; however, most studies use simple ‘gut malformations’ or ‘abnormal gut coiling’ categories for phenotypes. Given that gut phenotypes can be complex, the new ventral views and gut-coiling diagrams will help researchers understand digestive system development in the frog and will enable more accurate descriptions of gut phenotypes. Similarly, we predict this work will aid the staging of embryos for phenotype scoring for EAMA and AMA (Extended/Amphibian Metamorphosis Assay). EAMA and AMA assess the effects of potential endocrine and thyroid pathway disrupting substances on amphibian metamorphosis, by scoring limb phenotypes, abnormal behavior, time to metamorphosis, and mortality during prometamorphosis and climax metamorphosis stages (Dang, 2019; Ortego et al., 2021).

In modern times, normal tables of development have provided the basis for more systematic annotation of biological data using ontologies, controlled vocabularies that make data machine readable and thus enable computational analysis (Hunter et al., 2003; Van Slyke et al., 2014; Bard, 2012; Slater et al., 2020). In Xenbase, developmental stages, cell types, tissues and organs are represented in the *Xenopus* Anatomical Ontology (the XAO), with term definitions being directly adapted from the Normal Table (e.g. NF stage 42 is XAO ID:l1000054) (Segerdell et al., 2013). The XAO is used to describe gene expression patterns and is the foundation for the *Xenopus* Phenotype Ontology (the XPO), which is used to describe experimental phenotypes and link these phenotypes to human disease (Fisher et al., 2021). On Xenbase, the new Zahn drawings also illustrate the XAO pages for the NF stages, and researchers can explore stage-specific gene expression data. The online version of the Landmarks Table on Xenbase (https://www.xenbase.org/entry/landmarks-table.do) has live links that direct researchers to (1) gene pages, with links to extensive gene-specific expression data, genomic resources and orthologs, and to (2) XAO pages, with tissue-specific gene expression data (Figs S2 and S3). Researchers can use Xenbase search tools to interrogate and efficiently connect anatomical development to curated data (gene expression, phenotypes and associated diseases) and primary literature sources (James-Zorn et al., 2018). Importantly, this online version of the Normal Table will be a dynamic, evolving resource that can be updated with the latest molecular data attributed to different stages and tissues.

In summary, we hope that these new open-source online resources will encourage and enhance exploration of the classic Nieuwkoop and Faber Normal Table of *Xenopus* development. We anticipate that the new Zahn images and their integration on Xenbase will facilitate the ongoing renaissance of *Xenopus* research in the genomics of human disease modeling. Finally, we hope that releasing the Zahn images as an open-access resource will support educators teaching the next generation of embryology students and *Xenopus* researchers.

## MATERIALS AND METHODS

### Producing the illustrations

The method of producing this new set of illustrations began with observations of live specimens under a stereomicroscope, sketching and photographing specimens to produce reference images.

This process was essentially the same as that described previously (Zahn et al., 2017). Animals used in this project were wild-type J-strain *X. laevis* (NXR_0.0024), accessed at the National *Xenopus* Resource (NXR; RRID:SCR_013731) at the Marine Biology Laboratory (MBL) at Woods Hole, MA, USA. Animals were handled following the ARRIVE and NIH Guidelines for Use and Care of Laboratory Animals that were approved by the Institutional Animal Care and Use Committee of the MBL. Representative embryos from several different clutches were used. The developmental stages were prior to overt sexual identification. Vitelline membranes were removed from embryos at NF stages 13-15, because if not removed, the membrane flattens the neural plate, making it more difficult to tell NF stages 13-15 apart (Sive et al., 2007a). Likewise, the membranes were removed from stage NF 22-35 tailbud embryos before photographing to remove the curve in the body axis. Swimming tadpoles were anesthetized using approved methods. Specimens were photographed using a Zeiss steREO Discovery.V12 microscope with an AxioCam MRc camera. Larger tadpoles and froglets were imaged with an Apple iPhone XR camera.

Draft drawings were assessed by the authors (A.M.Z., S.A.M., P.D.V., D.R.B., D.S.A., N.M.N.-Y., J.G.), all experienced domain experts, to ensure that they illustrated stereotypical anatomical features, and additional reference images were all provided to the illustrator from our labs (A.M.Z., S.A.M., D.R.B., N.M.N.-Y.). Additional high-resolution reference images of staged embryos were kindly provided by the *Xenopus* community [e.g. the Willsey Laboratory (University of California, San Francisco, CA, USA) provided dorsal views of late-stage tadpoles showing brain morphology; and we used Kirschner Laboratory (Harvard Medical School, MA, USA) and other images posted in the *Xenopus* community Slack forum]. In addition to the fully shaded illustrations, simple line drawings and outlines for many of the stages were also drawn (Fig. S1). New diagrams of the gut coiling process (by J.G. and N.Z.) accompany ventral views of NF stages 41-46, illustrating the shape and directionality of this dynamic process. Gut coiling diagrams were based on anatomical studies of the *Xenopus* alimentary system, for which tadpoles were anesthetized and/or euthanized in accordance with North Carolina State University Institutional Animal Care and Use Committee regulations. Final versions of all illustrations were produced in a digital format using applications from Adobe's Creative Suite and a Wacom Cintiq Pro 32 digital drawing display. All drawings are provided as 300 dpi, high-resolution pixel-based art, and include a scale bar and, where applicable, a compass rose, indicating size and orientation, respectively.

### Accessing Zahn drawings on Xenbase

The Zahn drawings presented here and those from the XenHead project (Zahn et al., 2017) are available as individual digital files (jpg format) and as a compiled printable sheet (pdf format) on Xenbase in the Anatomy & Development module (http://www.xenbase.org/entry/zahn.do). Additionally, the Zahn drawings are used to illustrate the XAO pages (http://www.xenbase.org/anatomy/xao.do?method=display) for relevant NF stages (Fig. S2).

## Supplementary Material

Supplementary information

Reviewer comments

## Poster

Poster: *Xenopus* staging series

## Poster

A poster of the Zahn drawings showing key stages of *Xenopus* development is available as supplementary material in the online version of this article at https://journals.biologists.com/dev/article-lookup/DOI/10.1242/dev.200356.
