# Supplementary material for: Normal Table of Xenopus development: a new graphical resource
Source: Development. 2022 Jul 14;149(14):dev200356. doi: 10.1242/dev.200356 (PMC9445888; doi:10.1242/dev.200356)
Supplement: Supplementary information [file develop-149-200356-s1.pdf]

Figure S1

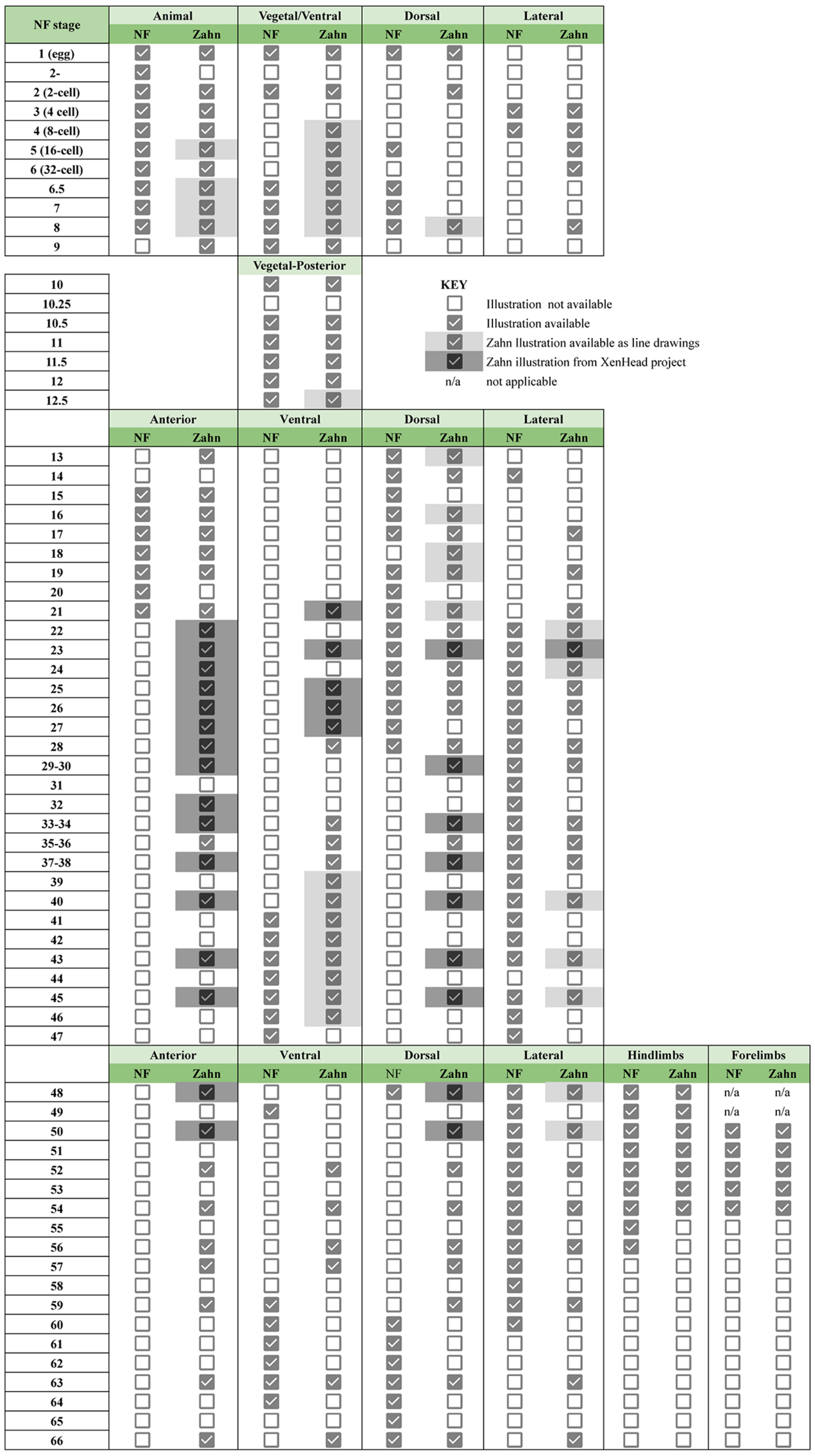

Fig. S1. A comparison of available illustrations of *Xenopus laevis* developmental stages from Nieuwkoop and Faber's Normal Table (1994 edition) and those by Natalya Zahn. Check boxes show drawings which illustrate each stage in the view indicated in column headers (i.e., animal vegetal/ventral, vegetal-posterior, anterior, ventral dorsal, lateral). Note that Nieuwkoop and Faber's NF stage 3 and NF stage 4 'dorso-lateral' views are recorded in the 'lateral' view column, and NF stage 13, 14 15 and 16 'post-dorsal' views are recoded here in the 'dorsal' view column. Light grey shaded cells indicate that line drawings (n= 56) are available for specified stage/view. Darkly shaded cells indicated drawings are the those commissioned from XenHead project (Zahn et al., 2017). Open box: drawing not available. n/a: not applicable. Total Nieuwkoop and Faber illustrations = 125, and total Zahn illustrations = 142 (plus an additional 56 simple line illustrations), when counting each limb bud as an individual drawing in both series. Many of the Zahn drawings (but not all) are available as both complex, fully shaded illustrations and as simple unshaded line drawings. All drawings are available on Xenbase, but note that only the Zahn drawings are open access, and can be reused using this citation: Illustration © 2021 Natalya Zahn, CC BY-NC 4.0.

Development • Supplementary information

Figure S2

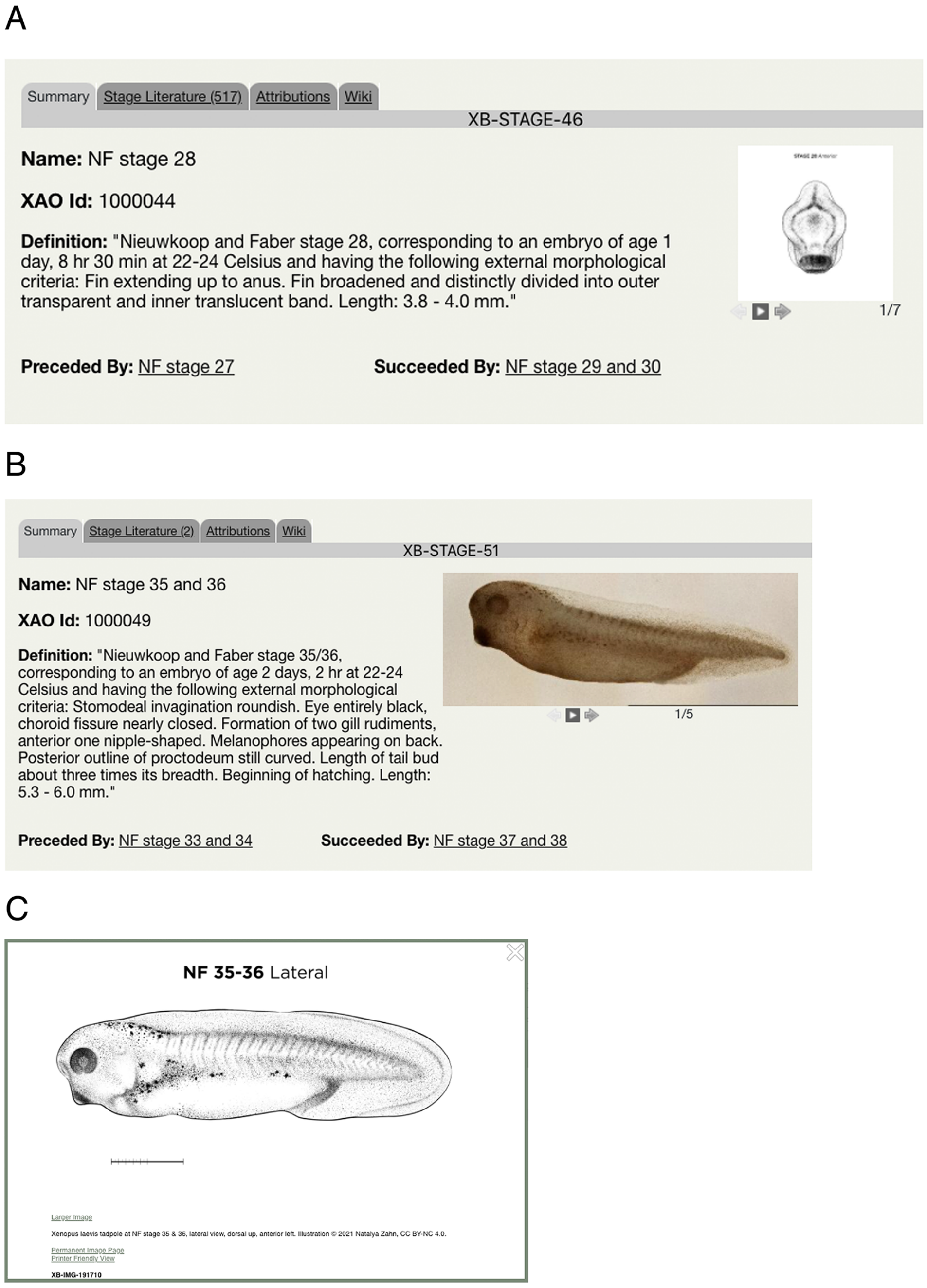

Fig. S2. Xenbase XAO pages for *Xenopus* embryo stages. Each XAO page for embryonic stages on Xenbase has a definition, based on the Nieuwkoop and Faber Normal Table, and is populated with Zahn drawings and other images (e.g., photographs from stereomicroscopy) when available. A. XAO page for NF stage 28. B. XAO page for NF stage 35 & 36, illustrated with a *Xenopus* community submitted image taken by Matthew Kofron (used here with their kind permission). C. Pop-up window from NF stage 35 & 36, activated by double click on the image, shows a larger view and has a left-right scroll arrow to view all images posted to this page, including Zahn drawings. Links from the pop-up will enlarge an image further or give researchers a printer friendly view.

Development • Supplementary information

Figure S3

A

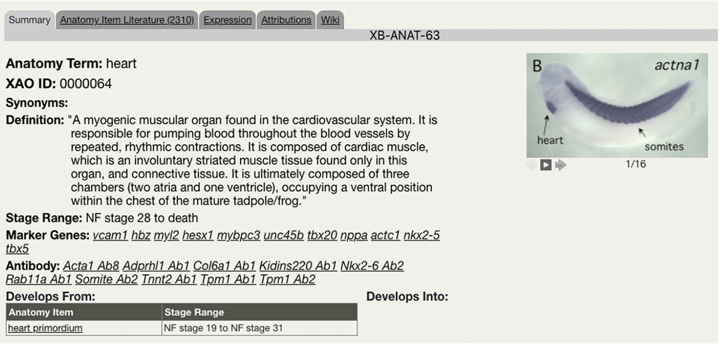
Summary | Anatomy Item Literature (2310) | Expression | Attributions | Wiki

XB-ANAT-63

Anatomy Term: heart

XAO ID: 0000064

Synonyms:

Definition: "A myogenic muscular organ found in the cardiovascular system. It is responsible for pumping blood throughout the blood vessels by repeated, rhythmic contractions. It is composed of cardiac muscle, which is an involuntary striated muscle tissue found only in this organ, and connective tissue. It is ultimately composed of three chambers (two atria and one ventricle), occupying a ventral position within the chest of the mature tadpole/frog."

Stage Range: NF stage 28 to death

Marker Genes: *vcam1 hbz myl2 hesx1 mybpc3 unc45b tbx20 nppa actc1 nkx2-5 tbx5*

Antibody: *Acta1 Ab8 Adprhl1 Ab1 Col6a1 Ab1 Kidins220 Ab1 Nkx2-6 Ab2 Rab11a Ab1 Somite Ab2 Tnnt2 Ab1 Tpm1 Ab1 Tpm1 Ab2*

Develops From:

Anatomy Item	Stage Range
heart primordium	NF stage 19 to NF stage 31

Develops Into:

B

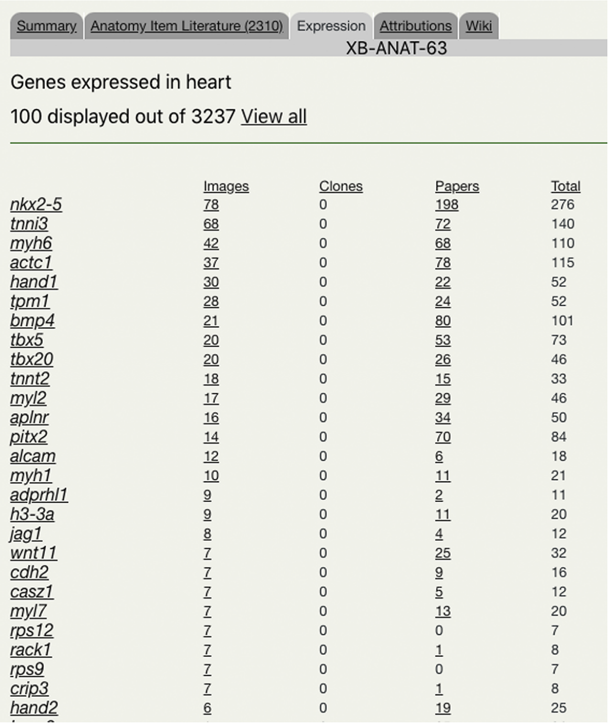
Summary | Anatomy Item Literature (2310) | Expression | Attributions | Wiki

XB-ANAT-63

Genes expressed in heart

100 displayed out of 3237 View all

	Images	Clones	Papers	Total
nkx2-5	78	0	198	276
tnni3	68	0	72	140
myh6	42	0	68	110
actc1	37	0	78	115
hand1	30	0	22	52
tpm1	28	0	24	52
bmp4	21	0	80	101
tbx5	20	0	53	73
tbx20	20	0	26	46
tnnt2	18	0	15	33
myl2	17	0	29	46
aplnr	16	0	34	50
pitx2	14	0	70	84
alcam	12	0	6	18
myh1	10	0	11	21
adprhl1	9	0	2	11
h3-3a	9	0	11	20
jag1	8	0	4	12
wnt11	7	0	25	32
cdh2	7	0	9	16
casz1	7	0	5	12
myl7	7	0	13	20
rps12	7	0	0	7
rack1	7	0	1	8
rps9	7	0	0	7
crip3	7	0	1	8
hand2	6	0	19	25
[illegible]	[illegible]	[illegible]	[illegible]	[illegible]

Fig. S3. Xenbase XAO anatomy terms pages. Anatomical entities (i.e., primary germ layers, cell types, tissues, organs, structures) are individual terms in the *Xenopus* anatomy ontology, the XAO. XAO terms used in the Landmarks Table link to the specific XAO page where information about that term is collated. A. XAO page (Summary tab) for heart (XAO:0000064), where the definition is based on Nieuwkoop and Faber's (1956) Normal Table criteria, and a curated list of molecular markers from Xenbase gene expression data are given. B. Expression tab of XAO page for heart collates all curated gene expression data. Columns are sortable based on number of primary sources, predominantly images (curated gene expression data from published research articles) and papers (where gene and anatomy are co-referenced). Click 'Images' heading to sort genes with the most curated gene expression figures from primary sources (and therefore the best candidates for molecular markers) at the top of the list.

Development • Supplementary information

Table S1. *Xenopus laevis* staging landmarks. For a live online Xenbase version see: https://www.xenbase.org/entry/landmarks-table.do

Developmental Process	NF stage number	NF stage name	LANDMARKS: EXTERNAL	LANDMARKS: INTERNAL	LANDMARKS: MOLECULAR MARKERS [*gene*: anatomy]
FERTILIZATION	n/a	mature egg	1 cell; ovulated unfertilized egg; animal hemisphere dark, vegetal hemisphere light; animal-vegetal axis in random orientation; soft texture	large nucleus, 'germinal vesicle' in animal hemisphere	***nanos1***: mitochondrial cloud; ***vegt***: vegetal cortex
	1	1-cell, fertilized egg	1 cell; vitelline membrane swells; eggs rotate with dark animal hemisphere up, light vegetal hemisphere down; firm texture; sperm entry point indictated by pigment condensation; germinal vesicle breakdown leaving pale spot in animal hemisphere	cleavage has not begun; germinal vesicle breaks down and pronuclei fuse; visible cortical layer thickest on animal and dorsal sides.	
CLEAVAGE	2-		first cleavage begins as a top to down furrow in animal hemisphere	cleavage furrow has not yet reached the vegetal pole	
	2	2-cell	2 cells; 1st cleavage plane is meridional along the dorso-ventral plane dividing right and left halfs		***sox3***, ***atp4a***: animal hemisphere; ***vegt***: vegetal hemisphere
	3	4-cell	4 cells; 2nd cleavage is meridional, perpendicular to the 1st cleavage; in many embryos, the 2 dorsal blastomeres (left and right) are smaller and lighter (on animal surface) than 2 larger darker ventral blastomeres (also left and right)[1]	cleavage cavity present	center
	4	8-cell	8 cells; 3rd cleavage plane is equatorial giving 4 smaller animal blastomeres (micromeres) and 4 larger vegetal blastomeres (macromeres); animal dorsal cells are lighter and ventral cells are darker in some embryos		***nanos1***: germ plasm; ***hwa***: dorsal Wnt signalling
	5	16-cell	16 cells; 4th cleavage is again meridional; animal blastomeres smaller than vegetal blastomeres; dorsal blastomeres lighter than ventral.		
	6	32-cell	32 cells; 5th cleavage equatorial, giving 4 rows each with 8 blastomeres; animal pole with smallest rosette of micromeres, 2 middle rows of irregular shaped cels, larger vegetal rosette of macromeres		
	6.5	morula	64 cells; 6th cleavage; cleavages becomming asynchronous; animal/dorsal blastomeres divide before vegetal blastomeres		***gdf1***: vegetal hemisphere; ***shroom1***, ***tfap2a***: animal hemisphere
BLASTULA	7	early blastula	128-512 cells; 7th-9th cleavages, no longer possible to count cells reliably; size of animal cells used to distinguish embryo stage	clear delineation of the 3 primary germ layer tissues; notochord begins to form from mediolateral convergence of dorsal presumptive axial mesoderm under the neural ectoderm	***nodal5 (nr5)***: earliest zygotic transcription
	8	middle blastula	~1000-4000 cells; 10-12+ cleavages; cell number no longer a stage guide; animal hemisphere with small dark pigmented cells refered to as 'animal cap'; animal surface looks 'pebbly'	at NF stage 8.5, after 12th cell divisions the mid-blastula transition (MBT) occurs; asynchronous internal cell division; zygotic genome activation	***gs17***, ***nr1***: major initiation of zygotic transcription
	9	late blastula	animal hemisphere still darkly pigmented; animal surface more like 'grains of sand' than 'pebbles' of NF stage 8	blastocoel now maximum size; 3 germ layers becoming distinct; ectoderm in the animal 1/3 of the embryo, a ring of mesoderm in the middle and yolky endoderm on the vegetal 1/3	***sox17a***: endoderm; ***tbxt***: mesoderm
GASTRULATION	10	initial gastrula	darker pigment from bottle cells on the dorsal vegetal surface indicate dorsal blastopore lip formation, between '11 & 1 o'clock'; blastopore groove where cell ingress, is not yet visible	initial formation of Spemann Organizer at the dorsal marginal zone/upper blaspore lip	***gsc***: upper blastopore lip; ***tbxt***: marginal zone
	10.25	early gastrula	blastopore lip extends laterally ~1/4 around the circumference, from ~ '10 to 2 o'clock' ; indentation of blastopore visible as a groove	marginal zone involutes on dorsal side; internally, dorsal endomesoderm begins to migrate anteriorly along bastocoel surface; vegetal rotation of endoderm mass	
	10.5	crescent-shaped blastopore	arch of pigmented blastpore lip extends laterally from doral to lateral sides ~1/4 to 1/2 around the circumference; yolk plug almost round	medial-lateral intercalation of dorsal mesoderm; 'Brachet's cleft' forms between internal leading edge endomesoderm and the dorsal mesoderm; neural induction begins, dorsal-ventral patterning	***chrd.1***, ***lhx1***: involuted dorsal mesoderm; ***ventx2.1***, ***wnt8a***: ventro-lateral marginal zone; ***hhex***: anterior endomesoderm
	11	horse-shoe shaped blastopore	pigmented blastopore lip ~ half the circumference, pigmented bottle cells extend to the ventral side; yolk plug ~1/2 diameter of embryo, is slightly elongated in the dorsal-ventral direction	Brachet's cleft begins to open forming the archenteron; ectoderm, mesoderm and endoderm germ layers specified; blastocoel becoming smaller, mesodermal mantle undergoes convergent extension and endoderm is internalized	***sox17a***: endoderm; ***tbxt***: mesoderm; ***sox2***: neurectoderm; ***krt12.4***: non-neural ectoderm
	11.5	large yolk plug	blastopore lip extends all the way around yolk plug; lower/ventral blastopore lip more darkly pigmented; yolk plug not quite round, ~1/3 of embryo diameter elongated in the dorsal-ventral direction	blastocoel displaced to the ventral side; involuting 'endomesoderm' mantle continues to extent anteriorly	
GASTRULATION / NEURULATION	12	medium yolk plug	areas of light and dark pigment radiating from yolk plug (flower petal like); yolk plug small and circular, a little less than 1/4 of egg diameter, diameter decreasing; neurectoderm transforming into the discernible neural plate	blastocoel begining to close as the archenteron expands	***sox2***, ***sox3***: neural plate
	12.5	small yolk plug	darker pigment lines on dorsal surface indicate future neural groove and neural plate; blastopore slit is slightly open, diameter decreasing and oval shaped	clear delineation of the 3 primary germ layer tissues; notochord forms from mediolateral convergence of dorsal axial mesoderm under the neural ectoderm	***chrd.1***, ***nog***, ***shh***: axial mesoderm, notochord
NEURULATION	13	slit blastopore	blastopore completely closed to a 'slit'; neural plate on dorsal side clearly outlined	neural crest form at the neural plate border, i.e., the boundary between the neural and non-neural ectoderm	***sox2***, ***sox3***: neural plate; ***ednra***, ***msx1***: neural plate border; ***snail2***; neural crest; ***tbx2***: cement gland primordium; ***myf5***: paraxial medoderm; ***rax***: optic field
	13.5	initial neural plate	sharp demarcation of anterior neural plate; anterior aspect of neural plate bent down; yolk plug internalized		***tubb2b***: differentating neural progenitors; ***msx1***: anterior neural plate border
	14	neural plate	neural plate obvious; dorsal midline thin with neural folds thickening anteriorly and laterally; neural folds begin to elevate; convergence extension begins to narrow neural plate posteriorly	blastocoel continues to close in the ventral foregut region as archenteron expands on the internal dorsal side	***pax6***: optic field, neural plate, lens placode; ***pax2***: between anterior and posterior neural plate
	15	early neural fold	neural folds distinct; anterior neural fold round; demarcation of neural plate clear caudally, narrowing in middle and caudal regions; neural groove deepens; pigmented cement gland primordium faintly visible at the ventral-anterior border of neural plate, cells stand out from epithelial layer.	physical segregation of cranial neural crest from anterior (pre-chordal) neural plate; blastocoel closes in the ventral foregut	***pax3***: neural fold/neural crest; ***pax8***: intermediate mesoderm; ***rax***, ***otx2***, ***six3***: optic field; ***hhex***: foregut endoderm
	16	mid-neural fold	eye primordia [2] become discernable as indentations near the lateral edges of the anterior neural plate; anterior neural plate 'rectangular' in shape; neural plate sharply constricted in the middle.	right and left cardiac mesoderm migrates to anterior-ventral midline; foregut diverticulum forms	***pax6***: optic field, anterior neural plate, and lens placode; ***nkx2-5***: cardiac progenitors; ***krt12.4***: ectoderm, non-neural ectoderm, epidermis
	17	late neural fold	anterior neural plate oblong, triangular, angles formed by eye primordia [2]; clear neurenteric canal along midline, posterior end of which continues over closed blastopore slit; neural folds closing in trunk region	delineation of cranial neural crest lateral to the anterior neural plate; 1st indication of somite segregation from presomitic mesoderm	***sox2***, ***sox3***: neural plate/neurenteric canal; ***otx2***, ***rax***, ***pax6***, ***six1***, ***sox2***: optic field; ***lhx1***: intermediate mesoderm
	18	neural groove	anterior part of neural plate narrow, club shaped; parallel neural folds very close but not touching	neural crest segregation begins; 3-4 anterior somites segregate from paraxial mesoderm	***myod1***: early somites and presomitic mesoderm; ***casz***: early somites; ***snai1***, ***egr2***, ***twist1***: neural crest; ***pax8***: otic placode.
	19	initial neural tube	neural folds moslty closed forming a neural tube, except for an open slit in the anterior neural plate; neurenteric canal deepening; dark pigmented cement gland primordium immediately ventral to anterior neural plate; laterla view of embryo convex oval, not elongated	migration of 4 neural crest streams begins; 4-6 anterior somites segregated	***ag1***, ***agr2***: cement gland primordium
	20	fused neural tube	neural tube fused anteriorly; no pharyngeal bulge visible; dark oval cement gland primordium below border of anterior neural plate; embryo starts to elongate; optic cup (eye primordium) forms; oral evagination (mouth) visible	neural crest extends to front to eye; paired glomus primordia present [2] ; anterior 6-7 somites; thickening of cardiac mesoderm	***rax***, ***pax6***, ***otx2***, ***six3***: optic vesicle; ***wt1***, ***rgn***: glomus; ***nkx2-5***: cardiac progenitor cells; ***egr2***: rhombomeres R3, R5, and neural crest; ***en2***: midbrain-hindbrain boundary; ***snai2***: neural crest; ***chrd.1***, ***nog***, ***ssh***: notochord
	21	neural tube	embryo has a dorsal curvature, with flat ventral surface; one pharyngeal arch bulge; neural tube completely closed; optic vesicle ('eyes') begin protuding, forming 2 oblique 'oval spots'; multiciliated cells form on the surface of the epidermis	8-9 somites; first indication of pronephric mesenchyme; otic placodes form posterior to optic vesicle	***pax8***, ***lim1***: pronephric mesenchyme; ***nrp1***: neural tube; ***pax3***: hatching gland; ***tuba4b***, ***cfap206***, ***foxj1***: multiciliated epidermal cell

Development • Supplementary information

EARLY ORGANOGENESIS		22	early tailbud	embryo begins to elongate (convergent extension); ventral surface slightly concaved; two pharyngeal arch bulges; distinct eye protrusion; anal opening displaced to ventral side	9-10 somites; ventral blood island forms; segreation of forebrain, midbrain and hindbrain		gsx1, sox3: brain segments; gata1, hba3, tal1: ventral blood island; nodal1: lateral plate mesoderm on the left side only; tbx6, foxd4l1.1: early tail bud
				EXTERNAL	INTERNAL	BEHAVIOR & PHYSIOLOGY	MOLECULAR MARKERS
		23	early tailbud	ventral surface concave giving embryo a 'coffee bean' look; two pharyngeal arch bulges; olfactory placodes thicken between eyes; slight depression of otic placode; jaw and gills separated by groove; "inverted Y shaped" hatching gland between eyes to cement gland	12 somites; forebrain regions telencephalon and diencephalon distinguishable		cxcl14, astl3a.1, pax3: hatching gland; myod1: somites and presomitic mesoderm; six1: olfactory placode; pax2, pax6, vax2: diencephalon; foxg1: telencephalon
		24	early tailbud	noticable elongation of the embryo and tail bud outgrowth; in dorsal view, eyes protruding out laterally less than gills; gill primordium area smooth (ungrooved)	15 somites; primary germ cells detecable in cell trunk endoderm; primary and secondary heart fields indictated in heart primordium	initial motor reactions to external stimuli	tbxt, lmo2, aplnr: tail bud; tnni3, nkx2-5: primary heart field; bmp4: secondary heart field; grip2, pgat: primordial germ cells; pcdh8.2: otic vesicle and tail bud; eya2: otic vesicle
		25	early tailbud	embryo still convex dorsally and concave ventrally; eyes protruding out laterally equal to or more than gills, gills now grooved; otic vesicle pigmented	16 somites; head somite 1 diminished; brain flexure ~90o	multi-cilliated cells in the epidermis become active	ag1: cement gland; tubb2b: brain and spinal cord
		26	tailbud	If liberated from the vitelline membrane the embryo is straight, not convex dorsally; if the embryo remains in the vitelline membrane it is curved laterally; tail bud obvious; otic (ear) vesicle protruding	17 somites; head somite I disintegrated; pronephros distinct; myotomes distinct	spontaneous movements begin	pax8, hnf1b, irx3: pronephric mesenchyme
		27	tailbud	tail bud defined in lateral view; fin translucent; lens begins to form, eyes flatten laterally; otic vesicle closes	heart fields merge forming a triangular-shape at ventral midline, behind the cement gland primordium and anterior to liver diverticulum; 19 somites		pax6, sox3, prox1, foxe3, nrl: lens; neurod1, sox3: epibranchial placodes
	ORGANOGENESIS	28	tailbud	tail bud elongates distally and extends downward to cloaca; fin divided into outer transparent (outer fin) and inner translucent bands (inner fin); black cement gland fully formed; otic vesicle separates from epidermis	heart primordium and pericardial cavity discernible; pronephric nephrostomes form; 20-22 somites; epibranchial placodes first segregate	embryos liberated from the vitelline membrane glide around due to multi-cilliated cell fluid flow	hand2, actc1: endocardial tube; dlx2, sox9, sox10: cranial neural crest; pax2, lhx1: nephrostomes; neurog2, eya1: epibranchial placodes; fgf8, sox9: otic vesicle
		29 & 30	late tailbud	tail bud distinct; outer fin edge transparent over entire length; gray disc of the eye cup now visible	23-25 somites segregated to end of tail; lumen in pronephric kidney collecting duct; appearance of glomus and thyroid; neural tube closure has formed the spinal cord		runx1: olfactory placode; lhx9: brain segments; foxa2, tubb2b: spinal cord; nphs1, wt1: glomus; nkx2-1: thyroid primordium; not: tail tip
		31	late tailbud	tail bud equal in length and height; nasal/olfactory pits first indicated	heart primordium extends ventrally and bends slightly to right; 22-23 post-otic somites; midbrain-hindbrain boundary distinct		hey1, myod1, actc1: somites; en2, pax2, fgf8: midbrain-hindbrain boundary
		32	late tailbud	tail bud ~1.5x longer than height; eye cup distinct, U-shaped (open); mouth primordium not visible	heart a linear tube with anterior outflow tract, left ventricle, atrioventricular canal and atrium; 26 post-otic somites; pronephric nephrostomes form; lung buds visible		pax2, vax1: optic stalk; cfap161: nephrostomes and multiciliated epidermal cells; nkx2-1: lung and thyroid progenitors; aldh1a3, agr2: otic vesicle
		33 & 34	late tailbud	tail bud ~2x longer than height; gut ~3 x longer than tail; eye cup open C-shape with darker pigmentation dorsally; mouth primordium a shallow vertical groove; 32 post-otic somites; pigmented cells (melanophores) first appear on head (near hindbrain) and anterior trunk (near pronephric kidney)	heart looping begins; heart beat clearly observable; foregut begin to constrict at trachea-eophagus boundary; cranial nerves distinct; thyroid primordium discernible [2]; pronephric kidney and duct formed with surrounding vasculature; thyroid primordium detectable		dab2: pronephric sinus, posterior cardinal vein; sox2: dorsal foregut; tubb2b: cranial nerves; myl2, bves: heart; actc1, mybpc3: heart and somites; pax2, lhx1: pronephric kidney and pronephric nephrostomes [2]
		35 & 36	free swimming tadpole [3]	tail bud ~3x longer than height; gut ~2 x longer than tail; outline of the proctodeum still curved; optic vesicle/retina completely black, choroidal fissure open; cardiac mesoderm starts to spontaneously contract; mouth invagination not quite round; 2 gill lobes; 36 post-otic somites; melanophores extend over top of head and along dorsal trunk	heart S-shaped, with distinct atrium lying dorsal to ventricle; vasculature to head and tail developing; liver bud visible posterior to heart; pronephric duct fused with rectal diverticulum, pronephric nephrostomes obvious	embryos naturally hatch from vitelline membrane to become free swimming	aplnr, hbz: blood vessels and heart; onecut, nr1h5, hhex: liver; aldh1a1: pronephric kidney/duct, choroidal fissure and olfactory bulb; tal1, hba3: ventral blood island; nkx3.2: mouth primordium
		37 & 38	free swimming tadpole	gut almost same length as tail; eye's choroid fissure closing ventrally but remain open; mouth invagination deep, round-shaped; heart contractions obvious, blood flow visible; proctodeum at obtuse angle (~140 degrees) to tail somites; 40 post-otic somites; melanophores extend over tail	paired lymph hearts; entire pronephric kidney functioning; ventral bud of pancreas formed;		tnni3: heart; sftpc: lung buds; myh6: lymph heart; atp1a1: pronephric kidney and pronephric duct; nkx2-1: thyroid and lungs; foxe3: thyroid and lens; ptf1a, pdia2: pancreatic buds
		39	free swimming tadpole	gut equal in length to tail somites; melanophores around nasal pits & along ventral edge of tail somites; ventral choroid fissure nearly closed; proctodeum at ~125 degree angle to tail somites; 43 post-otic somites	retinal ganglion cell axons reach optic tectum; mesonephric kidney begins to form		bmp4, hoxa13: proctodeum; map2, pou4f1; retinal ganglion cell layer; insm1, nos1: optic tectum
		40	free swimming tadpole	tail now longer than the abdomin; optic choroid fissure completely closed; mouth opening 'breaks through'; stomach and pancreas visible on the left side of gut; proctodeum at 90 degree angle to tail somites (lateral view), ~45 post-otic somites	gall bladder primordium formed and sometimes visible (irridescent on ventral view); blood circulation in gills visible	embryos begin taking gulps of air from the surface	cela1.2: pancreas; sfrp5, klf5: stomach; onecut, hhex, sox17a: gall bladder; hhex, nr1h5: liver
GUT-COILING		41	free swimming tadpole	conical shaped proctodeum formed, at angle of ~ 60 degrees to tail somites	myocardium thickens and develops trabeculae, atrium posterior to ventricle; torsion of gut starts; post-anal gut disappears; the pancreas, now visible in ventral view, posterior to left-sided stomach		clcnkb: pronephros; ins: pancreas; myod1: tail somites; s1pr1: brain segments; nkx2-5, tpm1: myocardium; hoxa13: proctodeum
		42	free swimming tadpole	opercular fold first visible; head somites I and II disappeared	trachea and esophagus seperate		sox2: esophagus and stomach; nkx2.1: trachea and lung buds; sftpc: lung buds
		43	free swimming tadpole	cement gland starts to lose pigmentation; lateral line pits visible	stomach has lengthened further; pancreas shifted to right side [5]; duodenum formed by 1st gut coil constriction to anterior-right; midgut and hindgut form hairpin curve, visible on left side (will become the 'apex' of future intestinal coil)		foxq1, spdef, bmpr1a, cfap161: stomach; cela1.2: pancreas; aldh1a2: duodenum;
		44	free swimming tadpole	heart fully formed and clearly visible; barbels/tentacles start to grow; gills/branchial basket shrinking	septum begins to form in cardiac atrium which is slightly anterior to ventricle; midgut and hindgut lengthened more; the intestinal apex visible in ventral view as a "U" shape in the upper left quadrant of the gut cavity	visual avoidance behavior begins	tnni3, frzb, sox9, nkx2-5: heart; cdx2: midgut-hindgut
		45	feeding tadpole [3]	operculum partly covers the gills, hindlimb bud not visible	midgut and hindgut continue to lengthen; the intestinal apex begins to rotate inward in a counterclockwise trajectory; spleen forms; mesonephric kidney	tadpoles to start swim continuously and begin feeding	darmin, a2m: midgut, hindgut and liver; nkx2-5: spleen primordium
	PREMETAMORPHOSIS	46	feeding tadpole	crescent-shaped hindlimb bud first appears although is difficult to see; pigment cells appear on eye and around abdomen; trunk somite 1 disappeared	midgut and hindgut lengthen further- apex continues to rotate inward, forming multiple coils of intestine; blood circulation to gills diminishing	food can be seen in intestine as now feeding	
		47	feeding tadpole	irredescent gold-coloured abdominal wall surrounds coiled gut; blood circulation visible from heart to gills, and through paired dorsal aorta; cement gland starts to degenerate; barbels/tentacles longer	retinal ganglion cells have formed complex synapses with optic tectum neurons; thyroid gland begins to function; thymus gland detectable		foxn1: thymus
		48		hindlimb bud now clearly visible, with nearly semi-circular shape	retinal ganglion cells-optic tectum synapses more compact		fgf8, spry1, sall4: hindlimb bud
		49		hindlimb bud length equal to it's width	thyroid follicles first appear		
		50		hindlimb bud slightly constricted at base; tiny oval forelimb buds just visible	gonads undifferentiated		hoxa13: forelimb bud; spry4: hindlimb bud
		51		hindlimb bud is cone-shaped; forelimb bud is oval shaped (in lateral view)	resorption vacuoles in thyroid follicles first appear		hoxd10, hoxa13, hoxa9: hindlimb bud
		52		hindlimb bud with slight 'wrist' indent; forelimb bud slightly constricted at base	5 complete coils of the intestine (internal and external coils)	regeneration competent [4]	sox9: hindlimb digits (cartilage elements)

Development • Supplementary information

	53		hindlimb bud paddle-like, with wrist constriction, hindlimb digits not discernable; forelimb bud with slight wrist constriction	onset of sexual differentiation of gonads	regeneration competent; athyroid animals have arrested development	
	54		hindlimb bud length (not including foot) 2x the width; foot paddle splayed with 5 digits and thinner inter-digital webbing; forelimb paddle with 4 digits and thinner inter-digital membranes	pronephric kidney begins to atrophy	thyroid hormone detectable in blood	***tbx4***, ***sall4***: interdigital mesenchyme
PROMETAMORPHOSIS	55		hindlimb length (not including foot) 3x width; forelimb hand rotates 90 degress, free parts of fingers as long as they are wide	all major muscles of hindlimb developed	regeneration restricted [4]	
	56		hindlimbs visible from above as they can rotate away from body; hindlimb length = ~ 5 tail somites; larval pigmentation pattern established	sexual differentiation of gonads into ovary or testis; hindlimb skeleton completey chondrified	regeneration restricted [4]	
	57		hindlimb length = ~ 9 tail somites; forelimb remains enclosed in operculum; lip folds form			
	58		hindlimb length = 11-12 tail somites; claws form on toes 1-3 (mostly always still white); forelimb emerges from operculum, elbows first; tail tip begins to atrophy	melanin/pigment deposited in under skin especially in tail	regeneration incompetent [4]	
	59		hindlimb muscular, claws start to harden and turn black, shortest toes first; finger tips reach base of hindlimb when forelimb is positioned along the abdomen; tentacles/barbels regress	melanin/pigment surrounds intersomitic blood vessels and between fibres of somites; forelimb muscles differentiated; pronephros no longer functional	regeneration incompetent [4]	
	60		gill chamber opening still wide; fingertips reach beyond base of hindlimb (almost to 'knee') when forelimb/arm is positioned along side of the abdomen; forelimb held posterior to heart; tail fins greatly reduced	pigmentation across body increases	regeneration incompetent [4]; animal switches from tail to leg swimming	
	61		first sign of gill resorption, openings to gill chamber much narrower; hindlimb and forelimb fully formed; forelimb at level of posterior half of heart	lateral finger-like protrusions from olfactory organ	cessation of feeding (due to oral and intestinal remodeling)	***obp***: olfactory organ
CLIMAX OF METAMORPHOSIS	62	tailed froglet	head slightly broader than abdomen; corner of mouth still infront of eye; forelimb reaches middle of heart; ventral tail fin gone from abdomen; adult skin on hindlimbs; only tiny nubs of barbels/tentacles remain.	tiny 'stirnorgan' (light detecting cells/part of pineal gland) appears; notochord atrophies along length of tail	peak levels of thyroid hormone in plasma	
	63	tailed froglet	head narrower than abdomen; barbels/tentacles (most often) completely gone; forelimb at level of anterior half of heart; tail shortens as tail somites are rapidly resorbed, tail still slightly longer than body			
	64		corner of mouth behind eye; tail length is about 1/3 of body length, at level of ankle when legs are in typical neutral position; body completely covered in adult skin, but 'border lines' clearly visible	thymus gland ventral-lateral to otic capsule		
	65		tail length a few millimeters, all tail somites have disappeared; body completely covered in adult skin, but 'border lines' still visible in some areas		feeding resumes	
	66	froglet	tail very nearly gone, not visible from ventral view; adult skin 'border lines' have disappeared, froglet body ~ 10mm long	skin remodelled with underlying dermis and secretory glands	thyroid hormone in plasma returns to prometamorphic levels	

FOOT NOTES

1 Dorsal-ventral pigment variation only occurs in some batches of embryos. Select 2-4-cell embryos with clear pigment variation - otherwise only accurate about 70% of time.
2 In the Normal Table and other texts, organ primordia are often called 'anlage'; they are visualized by as a thickening of specific cells via histology or by molecular markers. Search specific XAO terms on Xenbase for more molecular markers
3 NF stages 41-66 are not referred to by specific 'stage names' by Nieuwkoop and Faber
4 Regeneration classes from Aztekin et al 2021 PMID:34105722.
5 Left and right sides refer to that of the tadpole/embryo, and not the viewer.

Development • Supplementary information
