# Supplementary material for: Normal Table of Xenopus development: a new graphical resource
Source: Development. 2022 Jul 14;149(14):dev200356. doi: 10.1242/dev.200356 (PMC9445888; doi:10.1242/dev.200356)
Supplement: Poster: Xenopus staging series [file develop-149-200356-s2.pdf]

Development

The Zahn drawings: a new graphical resource of the Normal Table of *Xenopus* development

Illustrations of embryonic development from fertilized egg, through cleavage, gastrulation, neurulation, tailbud growth, tadpole, metamorphosis and froglet stages.

Natalya Zahn, Christina James-Zorn, Virgilio G. Ponferrada, Dany S. Adams, Julia Grzymkowski, Daniel R. Buchholz, Nanette M. Nascone-Yoder, Marko Horb, Sally A. Moody, Peter D. Vize and Aaron M. Zorn

The Company of Biologists

Cleavage

Dorsal (D) and ventral (V) sides of cleavage stages are indicated by compass directions (D-V)

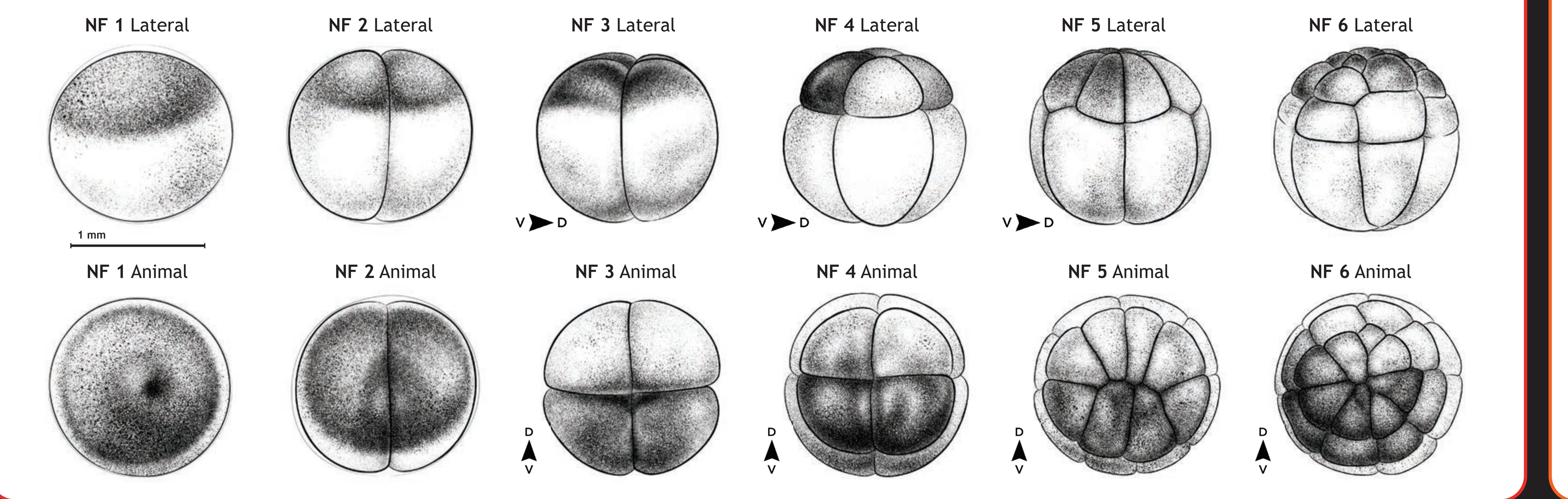

Blastula

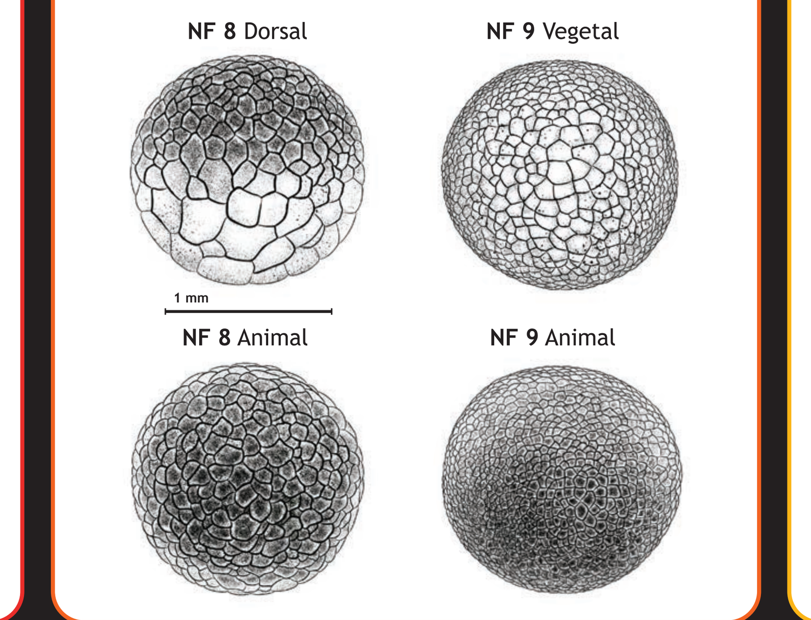

Gastrula

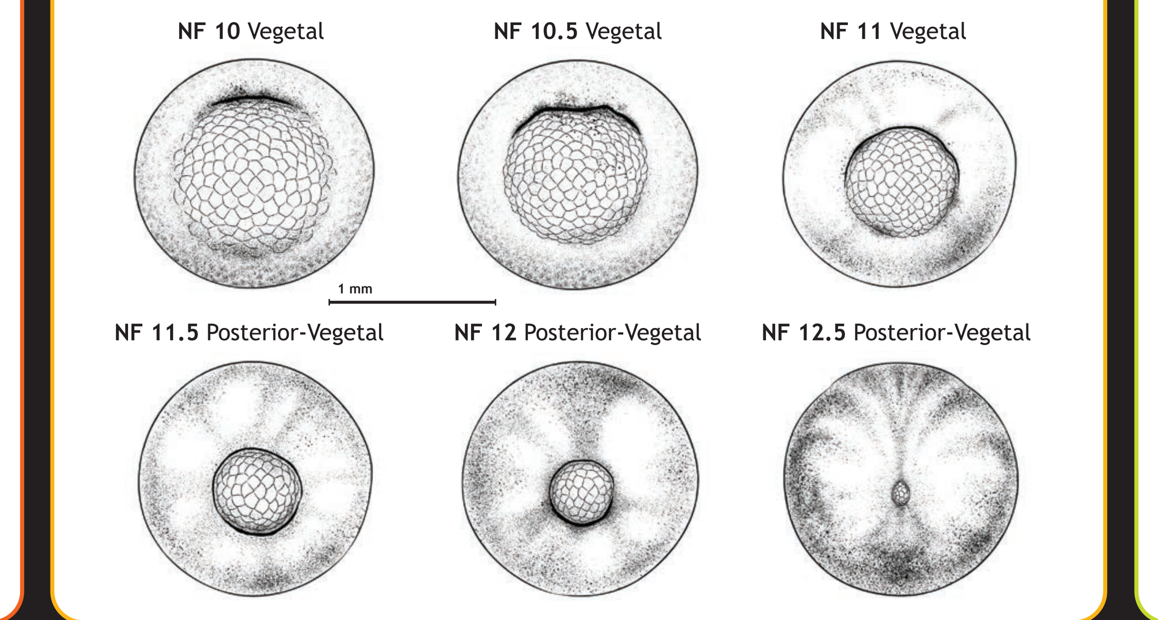

Neurula

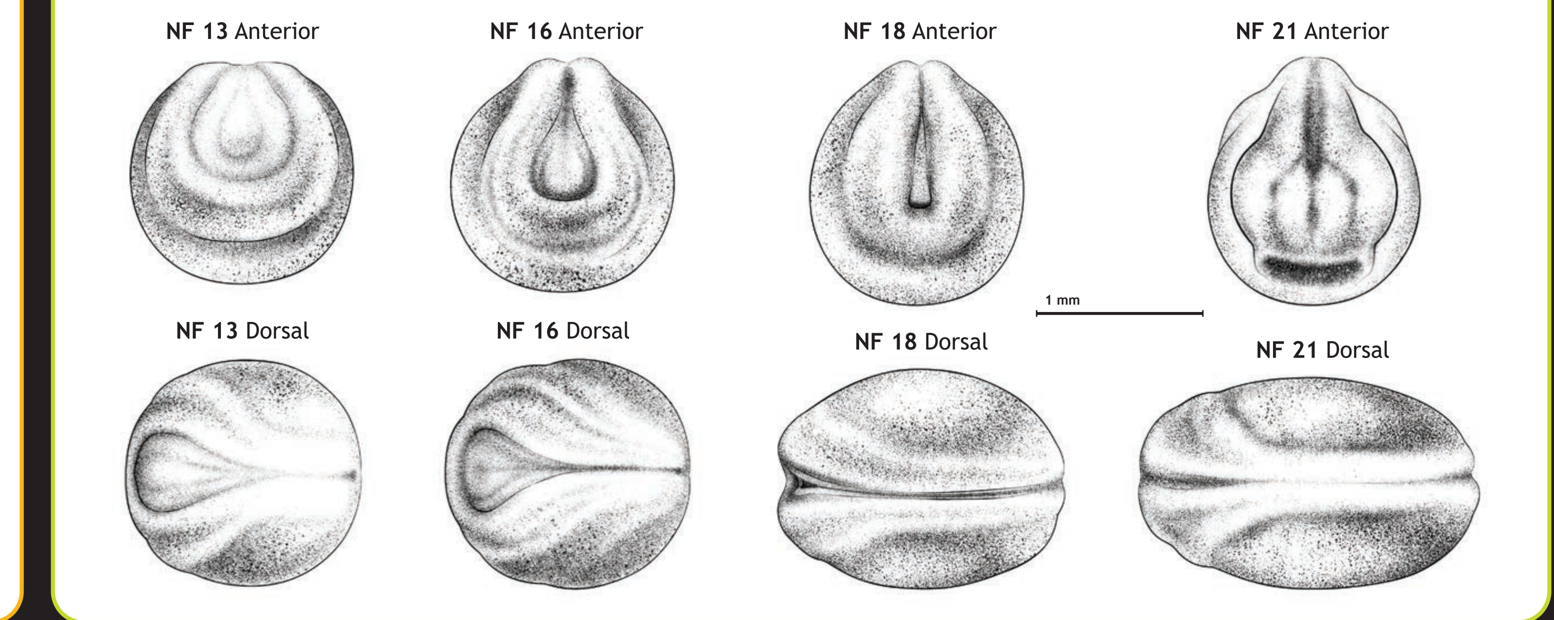

Early tailbud

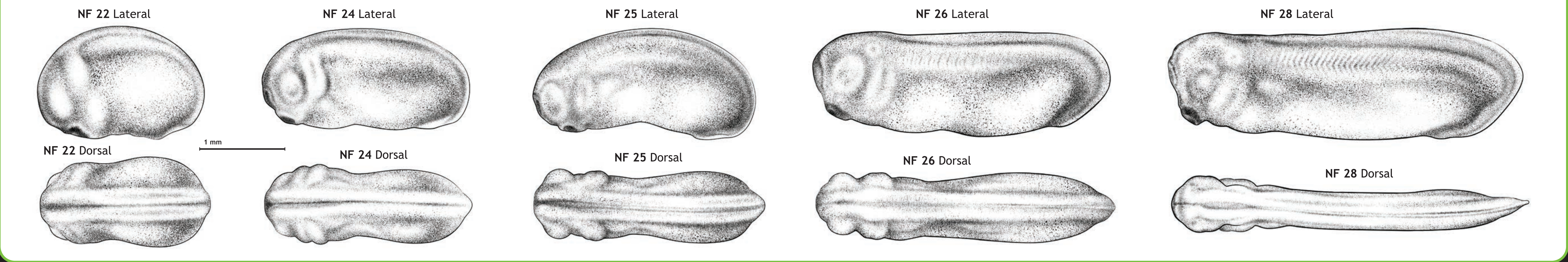

Late tailbud

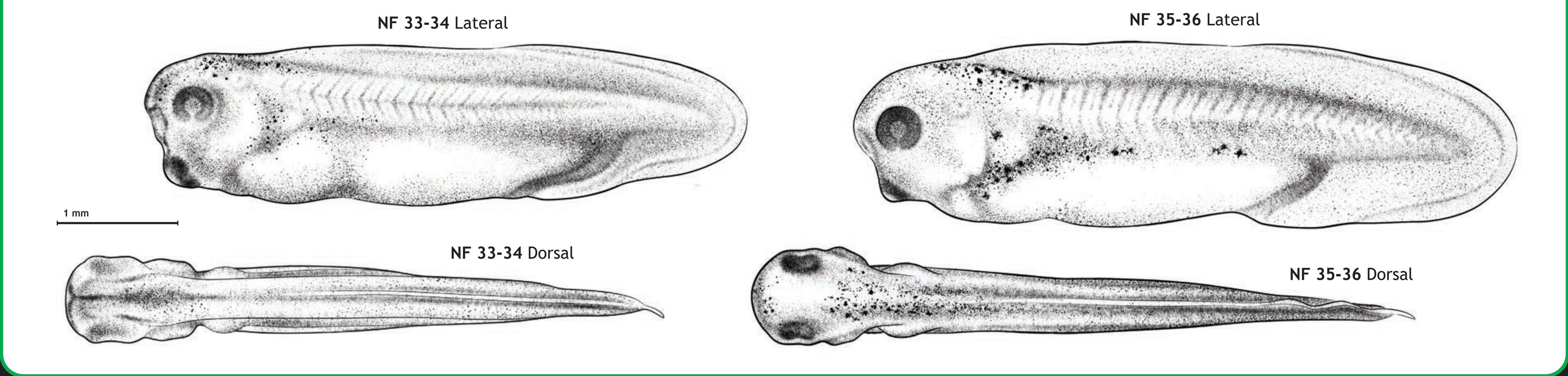

Late tailbud

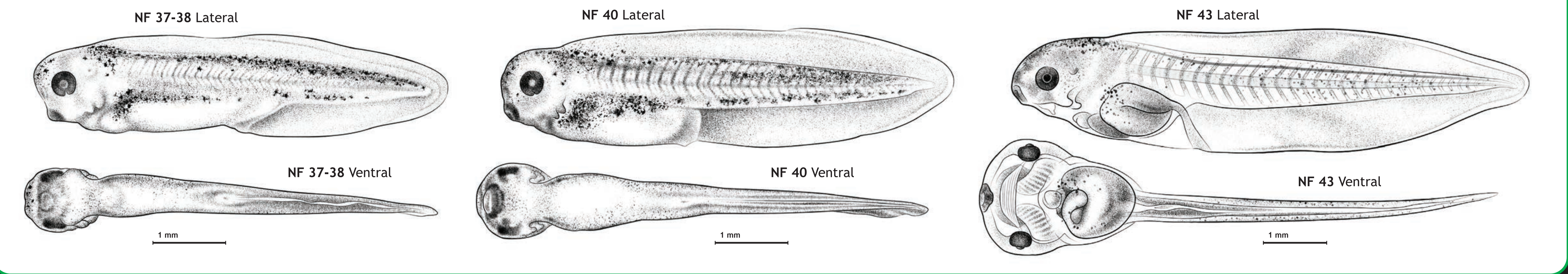

Premetamorphic tadpole

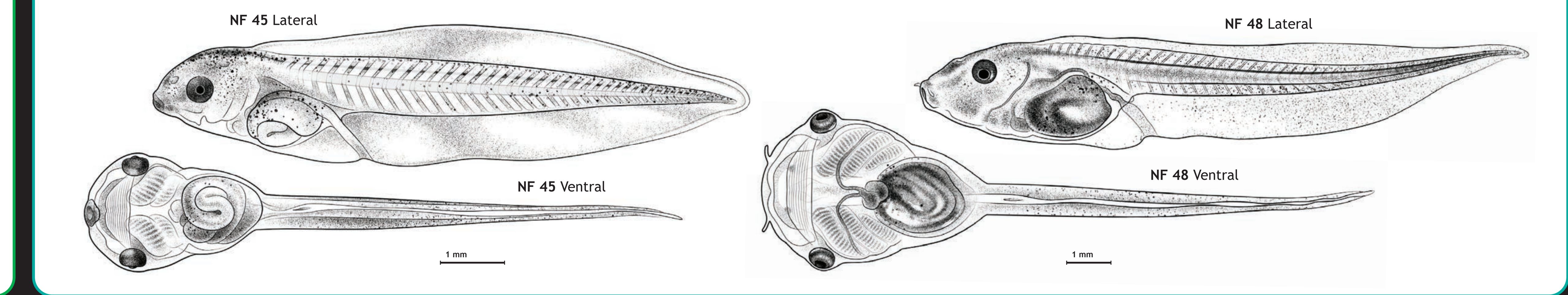

Metamorphic tadpole and froglet

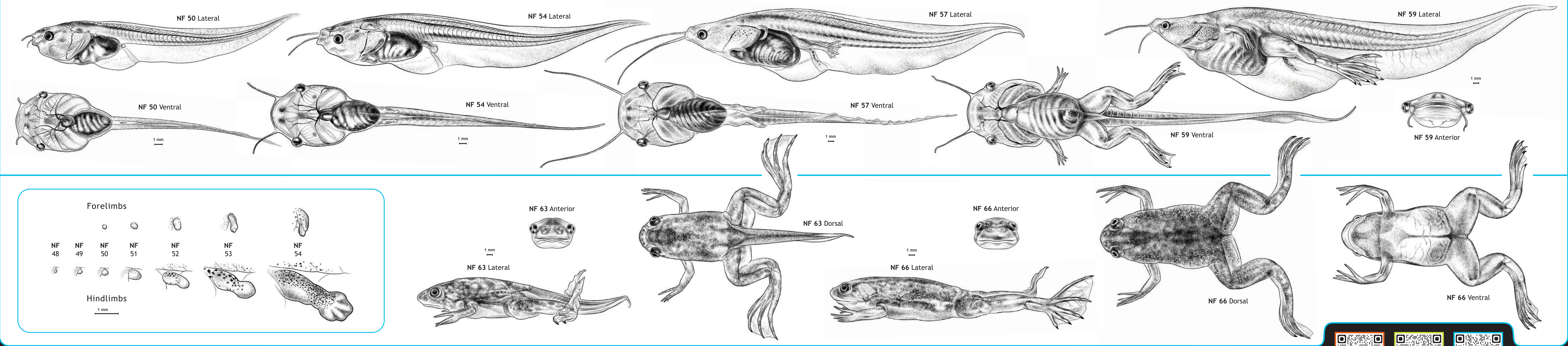

These images are to be used in conjunction with the original Nieuwkoop and Faber text, describing the anatomical transitions of *Xenopus* development (see *'Normal table of Xenopus laevis (Daudin): a systematical and chronological survey of the development from fertilized egg till the end of metamorphosis.'* 1956, 1994).

The full set of 130 drawings, including additional views and stages, can be found on Xenbase (https://www.xenbase.org). See the associated Landmarks Table for key morphological features and marker gene expression that quickly and reliably distinguish *Xenopus* embryonic stages (visit https://www.xenbase.org/entry/landmarks-table.do). All drawings are available for use by the research and academic community under a CC-BY-NC 4.0 license.

Poster ©2022, published by The Company of Biologists Ltd. doi: 10.1242/dev.200356
Drawings ©Natalya Zahn, available for research and academic use under a CC-BY-NC 4.0 license
Scan QR codes to access: the Zahn drawings on Xenbase; the Landmarks Table on Xenbase; or the article at Development.
