# Supplementary material for: Normal Table of Xenopus development: a new graphical resource
Source: Development. 2022 Jul 14;149(14):dev200356. doi: 10.1242/dev.200356 (PMC9445888; doi:10.1242/dev.200356)
Supplement: Reviewer comments [file develop-149-200356_review_history.pdf]

Normal table of *Xenopus* development: a new graphical resource

Natalya Zahn, Christina James-Zorn, Virgilio G. Ponferrada, Dany S. Adams, Julia Grzymkowski, Daniel R. Buchholz, Nanette M. Nascone-Yoder, Marko Horb, Sally A. Moody, Peter D. Vize and Aaron M. Zorn
DOI: 10.1242/dev.200356

Editor: Steve Wilson

Review timeline

Original submission:	18 November 2021
Editorial decision:	18 January 2022
First revision received:	11 March 2022
Accepted:	17 March 2022

Original submission

First decision letter

MS ID#: DEVELOP/2021/200356

MS TITLE: Normal Table of Xenopus development: a new graphical resource.

AUTHORS: Natalya Zahn, Christina H James-Zorn, Virgilio G Ponferrada, Dany S Adams, Julia K Grzymkowski, Daniel R Buchholz, Nanette M. Nascone-Yoder, Marko Horb, Sally A Moody, Peter D Vize, and Aaron M Zorn

I have now received all the referees reports on the above manuscript, and have reached a decision. The referees' comments are appended below, or you can access them online: please go to BenchPress and click on the 'Manuscripts with Decisions' queue in the Author Area.

The overall evaluation is positive and we would like to publish a revised manuscript in Development after you have considered the suggestions from the referees. Please attend to these comments in your revised manuscript and detail them in your point-by-point response.

Reviewer 1

Advance summary and potential significance to field

This article describes a new resource of high quality images of different stages of development of Xenopus laevis, from Stage 1 fertilized egg (Stage 1) through to the completion of metamorphosis (Stage 66). The images are available on Xenbase and are downloadable and can be used for research and teaching purposes, as long as their use is appropriately attributed, including referencing this publication.
As such, this paper does not provide any new information, but rather provides a new step of attributable images for staging Xenopus embryos, given that permission for the use of the original images published by Nieuwkoop and Faber can no longer be obtained (authors are now deceased and publisher is no longer in business). In summary, this article provides a very useful resource for the developmental biology community, in particular the Xenopus community. For this reason I enthusiastically recommend that this resource should be published in Development.

© 2022. Published by The Company of Biologists under the terms of the Creative Commons Attribution License (https://creativecommons.org/licenses/by/4.0/).

Comments for the author

Minor Comment:
This resource of images are valuable, but it will be important that the community continues to use (and read) the original Nieuwkoop and Faber publication, as it contains much more than simply a series of external images of the embryos at different stages. In fact the staging system of Nieuwkoop and Faber was not just based on the external features of the embryos, but mostly, they were based on internal features, based on histology. However, their publication did not include the actual histological data; only a description of the histological data. This was partly addressed (but only until Stage 27) by the beautiful publication in 1991 of Hausen and Riebesell in their book, "The Early Development of Xenopus laevis: An Atlas of the Histology" by Springer-Verlag (publisher). I find it a bit odd that this publication fails to reference the histological work of Hausen and Riebesell, which complements very nicely not only the Nieuwkoop and Faber publication, but also this publication. In the end, a full series of not only the external features of Xenopus laevis, but also their internal features, via histology, would be a wonderful resource for research and teaching communities.
But this, of course, is beyond the scope of this article. Something perhaps to mention and encourage others to pursue in the future, alongside other descriptive works, such as transcriptomic, proteomic, metabolomic studies.

Reviewer 2

Advance summary and potential significance to field

Zahn and colleagues have provided fantastic new resource for the Xenopus community and I'm very pleased to strongly support publication in Development.
Not only are the drawings exceptional, but the new Supp. Table provides an equally exceptional resource for researchers, educators, and students.
Moreover, the paper is very comprehensively written, providing not only a framework for the drawings but also assiduously linking the morphological representations to known patterns of gene expression (something that incidentally, might have annoyed Nieuwkoop himself, as the authors might recall from '94…).

Regardless, this is truly excellent new resource, and I propose only very minor modifications, which the authors should be given the liberty to ignore.

Comments for the author

1. Given the excellent historical overview in the first paragraph, the authors may wish to cite Hopwood's short history of Normal Tables in IJDB:
A history of normal plates, tables and stages in vertebrate embryology Int J Dev Biol. 2007;51(1):1-26. doi: 10.1387/ijdb.062189nh.
Nick Hopwood
2. I found the very long paragraph starting on line 280 to be daunting.
Perhaps split it up? Or even consider a sub-heading about internal criteria.
3. In fig. 2, i wonder why st. 6.5 and 7 are not rendered with pigment in the animal view as the other stages are. The uninitiated might find this confusing.
This may be for technical reasons, but if so, it should be clearly stated in the Legend that the lack of pigment is due to line drawing as opposed to rendering.
4. Also in figure 2, the drawing makes the equatorial blastomeres appear larger than the vegetal pole blastomeres. Is this quite right? I confess I don't often look at this stage, so it may be. But if so it is surprising and might be noted in the legend.
5. It will be a crime if Development does not put out a poster with these drawings. Doing so would ensure that decades from now crusty old copies -still proudly displaying the Development logo- will be coveted talismans.

© 2022. Published by The Company of Biologists under the terms of the Creative Commons Attribution License (https://creativecommons.org/licenses/by/4.0/).

Reviewer 3

Advance summary and potential significance to field

This is an unusual submission to Development but one of potentially great significance to the Xenopus community. Accurate staging of Xenopus embryos is now all the more important with the advent of studies like the single cell atlas where matching developmental stages in different experimental contexts is paramount.
The Introducting ives a very interesting overview of the original Normal Table and historical context that should be of wide interest to frog biologists. It also highlights some of the limitations of the current Table both anatomically and in terms of Copyright.
The next section contains a very good high level view of developmental stages and a discussion of useful markers. I don't know of anywhere else where these markers are described together.
Overall, the pictures are both beautiful and accurate.
The discussion also explains integration with the community resource "Xenbase", which will be useful.
There is also a pleasing collection of Xenopus anatomical papers referenced, which will undoubtedly be a very useful resource.
Overall, this manuscript is written in a clear and accessible way and should be of tremendous use to those new to Xenopus and "old timers". It is an important tool for an organism that provides the foundation for a surprisingly large amount of what we know about development. Thus, it is fitting that this highly useful and likely highly cited resource should be published in Development, a journal for the community supported by the community, and should provide it with the widest possible visibility for the manuscript which it deserves.
Anna Philpott, Professor of Cancer and Developmental Biology.

Comments for the author

Minor comments for consideration:
It is indeed important to note the effect of the vitelline membrane on morphology/staging at early neural stages, as they do, but the authors might also like to discuss the benefits (or otherwise) of removing the membrane at later neural stages (e.g. to allow the embryo to uncurl). It is possible to include some handy experimental tips on how to remove the vitelline membrane without damage to the embryo particularly early neurula as it can be tricky?
Some parts e.g. stages 29-38 might benefit from additional paragraph breaks to improve readability.

First revision

Author response to reviewers' comments

Response to reviewers:

We were pleased that the reviewers appreciated the value of this resource and that they were very supporting. We also thank the reviewers for their helpful suggestions on potential modifications and clarification to the text. We made all the suggested changed (in blue text), which we agree have improved the document. We hope you now find this acceptable for publication.

Reviewer 1 Advance Summary and Potential Significance to Field:

This article describes a new resource of high quality images of different stages of development of Xenopus laevis, from Stage 1 fertilized egg (Stage 1) through to the completion of metamorphosis (Stage 66). The images are available on Xenbase and are downloadable and can be used for research and teaching purposes, as long as their use is appropriately attributed, including referencing this publication. As such, this paper does not provide any new information, but rather provides a new step of attributable images for staging Xenopus embryos, given that permission for the use of the original images published by Nieuwkoop and Faber can no longer be obtained

© 2022. Published by The Company of Biologists under the terms of the Creative Commons Attribution License (https://creativecommons.org/licenses/by/4.0/).

(authors are now deceased and publisher is no longer in business). In summary, this article provides a very useful resource for the developmental biology community, in particular the Xenopus community. For this reason I enthusiastically recommend that this resource should be published in Development.

We are grateful that the reviewer appreciated the value of this resource and for their helpful suggestions, which we completely agree with.

Reviewer 1 Comments for the Author:
Minor Comment:

This resource of images are valuable, but it will be important that the community continues to use (and read) the original Nieuwkoop and Faber publication, as it contains much more than simply a series of external images of the embryos at different stages.

We agree. It has always been our intention that our image resource be used in conjunction with the original Nieuwkoop and Faber text, which contains a more extensive information. We have modified the text to emphasize this point more strongly.

In fact the staging system of Nieuwkoop and Faber was not just based on the external features of the embryos, but mostly, they were based on internal features, based on histology. However, their publication did not include the actual histological data; only a description of the histological data. This was partly addressed (but only until Stage 27) by the beautiful publication in 1991 of Hausen and Riebesell in their book, "The Early Development of Xenopus laevis: An Atlas of the Histology" by Springer-Verlag (publisher). I find it a bit odd that this publication fails to reference the histological work of Hausen and Riebesell, which complements very nicely not only the Nieuwkoop and Faber publication, but also this publication. In the end, a full series of not only the external features of Xenopus laevis, but also their internal features, via histology, would be a wonderful resource for research and teaching communities. But this, of course, is beyond the scope of this article. Something perhaps to mention and encourage others to pursue in the future, alongside other descriptive works, such as transcriptomic, proteomic, metabolomic studies.

We agree that the Hausen and Riebesell histology text is also a critical resource that complements Nieuwkoop and Faber and our new resource. We thank the reviewer for pointing out our oversight in not discussing this. We have now added text to the introduction and discussion, emphasizing out the value of the histology images from the Hausen text and how they are in fact also available on Xenbase, enabling users to compare external and internal anatomy in conjunction with the Xenopus Anatomy Ontology.

Reviewer 2 Advance Summary and Potential Significance to Field:

Zahn and colleagues have provided a fantastic new resource for the Xenopus community and I'm very pleased to strongly support publication in Development. Not only are the drawings exceptional, but the new Supp. Table provides an equally exceptional resource for researchers, educators, and students. Moreover, the paper is very comprehensively written, providing not only a framework for the drawings but also assiduously linking the morphological representations to known patterns of gene expression (something that, incidentally, might have annoyed Nieuwkoop himself, as the authors might recall from '94…).

Regardless, this is truly excellent new resource, and I propose only very minor modifications, which the authors should be given the liberty to ignore.

We thank the reviewer for appreciating the resource and for the helpful suggestions, which we agree with.

Reviewer 2 Comments for the Author:

1. Given the excellent historical overview in the first paragraph, the authors may wish to cite Hopwood's short history of Normal Tables in IJDB:

© 2022. Published by The Company of Biologists under the terms of the Creative Commons Attribution License (https://creativecommons.org/licenses/by/4.0/).

A history of normal plates, tables and stages in vertebrate embryology Int J Dev Biol. 2007;51(1):1-26. doi: 10.1387/ijdb.062189nh.
Nick Hopwood

Yes good suggestion for additional reading on the historical background. We have added this reference.

2. I found the very long paragraph starting on line 280 to be daunting. Perhaps split it up? Or even consider a sub-heading about internal criteria.

Agreed. We have split this up to make it clearer, discussing on internal criteria/anatomical systems separately.

3. In fig. 2, I wonder why st. 6.5 and 7 are not rendered with pigment in the animal view as the other stages are. The uninitiated might find this confusing. This may be for technical reasons, but if so, it should be clearly stated in the Legend that the lack of pigment is due to line drawing as opposed to rendering.

We have clarified in the figure legend that these images are examples of line drawings, not embryos without pigment.

4. Also in figure 2, the drawing makes the equatorial blastomeres appear larger than the vegetal pole blastomeres. Is this quite right? I confess I don't often look at this stage, so it may be. But if so it is surprising and might be noted in the legend.

In the lateral view of NF stage 8, the vegetal cells are seen obliquely, so they appear smaller than they really are. This is correct, as when viewed from vegetal surface they larger size is obvious.

5. It will be a crime if Development does not put out a poster with these drawings. Doing so would ensure that decades from now crusty old copies -still proudly displaying the Development logo- will be coveted talismans.

We completely agree and have always hoped that *Development* would produce a poster as well as use the cover image. This would be a wonderful advertisement at the next 2023 International Xenopus meeting which the senior author is co-organizing. We will pursue this option with the editor.

Reviewer 3 Advance Summary and Potential Significance to Field:

This is an unusual submission to Development but one of potentially great significance to the Xenopus community. Accurate staging of Xenopus embryos is now all the more important with the advent of studies like the single cell atlas where matching developmental stages in different experimental contexts is paramount.

The Introduction gives a very interesting overview of the original Normal Table and historical context that should be of wide interest to frog biologists. It also highlights some of the limitations of the current Table both anatomically and in terms of Copyright. The next section contains a very good high level view of developmental stages and a discussion of useful markers. I don't know of anywhere else where these markers are described together. Overall, the pictures are both beautiful and accurate. The discussion also explains integration with the community resource "Xenbase", which will be useful. There is also a pleasing collection of Xenopus anatomical papers referenced, which will undoubtedly be a very useful resource.

Overall, this manuscript is written in a clear and accessible way and should be of tremendous use to those new to Xenopus and "old timers". It is an important tool for an organism that provides the foundation for a surprisingly large amount of what we know about development. Thus, it is fitting that this highly useful and likely highly cited resource should be published in Development, a journal for the community supported by the community, and should provide it with the widest possible visibility for the manuscript, which it deserves.

© 2022. Published by The Company of Biologists under the terms of the Creative Commons Attribution License (https://creativecommons.org/licenses/by/4.0/).

Anna Philpott, Professor of Cancer and Developmental Biology.

Anna, thank you for appreciating the value of the work.

Reviewer 3 Comments for the Author:
Minor comments for consideration:
It is indeed important to note the effect of the vitelline membrane on morphology/staging at early neural stages, as they do, but the authors might also like to discuss the benefits (or otherwise) of removing the membrane at later neural stages (e.g. to allow the embryo to uncurl). It is possible to include some handy experimental tips on how to remove the vitelline membrane without damage to the embryo particularly early neurula as it can be tricky?

Good point, we will stress the importance of removing the vitelline membrane at later stages and have cited the Cold Spring Harbor protocols manual for methods of removing the membrane.

Some parts e.g. stages 29-38 might benefit from additional paragraph breaks to improve readability.

In retrospect we agree this part was a difficult read. We have split this up and edited for clarity.

Second decision letter

MS ID#: DEVELOP/2021/200356

MS TITLE: Normal Table of Xenopus development: a new graphical resource.

AUTHORS: Natalya Zahn, Christina H James-Zorn, Virgilio G Ponferrada, Dany S Adams, Julia K Grzymkowski, Daniel R Buchholz, Nanette M. Nascone-Yoder, Marko Horb, Sally A Moody, Peter D Vize, and Aaron M Zorn
ARTICLE TYPE: Techniques and Resources Article

I am happy to tell you that the referees are happy with your revisions and your manuscript has been accepted for publication in Development, pending our standard ethics checks. The referee reports are appended below. As you will be aware, Katherine is currently looking into producing a poster to be published alongside the paper.

Reviewer 1

Advance summary and potential significance to field

The revised manuscript is much improved and I now recommend acceptance of the revised manuscript.

Comments for the author

N/A

Reviewer 2

Advance summary and potential significance to field

New normal table pictures!

Comments for the author

This is an excellent resource.
Thank you to the authors!

© 2022. Published by The Company of Biologists under the terms of the Creative Commons Attribution License (https://creativecommons.org/licenses/by/4.0/).
